# Novel NSAID-Derived Drugs for the Potential Treatment of Alzheimer’s Disease

**DOI:** 10.3390/ijms17071035

**Published:** 2016-06-30

**Authors:** Ivana Cacciatore, Lisa Marinelli, Erika Fornasari, Laura S. Cerasa, Piera Eusepi, Hasan Türkez, Cristina Pomilio, Marcella Reale, Chiara D’Angelo, Erica Costantini, Antonio Di Stefano

**Affiliations:** 1 Department of Pharmacy, University “G. d’Annunzio” of Chieti-Pescara, Via dei Vestini 31, 66100 Chieti Scalo (CH), Italy; ivana.cacciatore@unich.it (I.C.); l.marinelli@unich.it (L.M.); e.fornasari@unich.it (E.F.); l.cerasa@unich.it (L.S.C.); piera.eusepi@unich.it (P.E.); hasanturkez@gmail.com (H.T.); cristina.pomilio@dompe.it (C.P.); 2 Department of Molecular Biology and Genetics, Erzurum Technical University, Erzurum 25240, Turkey; 3 Dompé Farmaceutici S.p.A.,Via Campo di Pile, 67100 L’Aquila (AQ), Italy; 4 Department of Medical Oral and Biotechnological Sciences, University “G. d’Annunzio” of Chieti-Pescara, Via dei Vestini 31, 66100 Chieti Scalo (CH), Italy; marcella.reale@unich.it (M.R.); chiara.dangelo@unich.it (C.D.); erica.costantini@unich.it (E.C.)

**Keywords:** anti-inflammatory drugs, Alzheimer’s disease, lipoic acid, neuroinflammation

## Abstract

Nonsteroidal anti-inflammatory drugs (NSAIDs) have been suggested for the potential treatment of neurodegenerative diseases, such as Alzheimer’s disease (AD). Prolonged use of NSAIDs, however, produces gastrointestinal (GI) toxicity. To overcome this serious limitation, the aim of this study was to develop novel NSAID-derived drug conjugates (Anti-inflammatory-Lipoyl derivatives, **AL4**–**9**) that preserve the beneficial effects of NSAIDS without causing GI problems. As such, we conjugated selected well-known NSAIDs, such as (*S*)-naproxen and (*R*)-flurbiprofen, with (*R*)-α-lipoic acid (LA) through alkylene diamine linkers. The selection of the antioxidant LA was based on the proposed role of oxidative stress in the development and/or progression of AD. Our exploratory studies revealed that **AL7** containing the diaminoethylene linker between (*R*)-flurbiprofen and LA had the most favorable chemical and in vitro enzymatic stability profiles among the synthesized compounds. Upon pretreatment, this compound exhibited excellent antioxidant activity in phorbol 12-miristate 13-acetate (PMA)-stimulated U937 cells (lymphoblast lung from human) and Aβ(25–35)-treated THP-1 cells (leukemic monocytes). Furthermore, **AL7** also modulated the expression of COX-2, IL-1β and TNF-α in these cell lines, suggesting anti-inflammatory activity. Taken together, **AL7** has emerged as a potential lead worthy of further characterization and testing in suitable in vivo models of AD.

## 1. Introduction

Nowadays, non-steroidal anti-inflammatory drugs (NSAIDs) are widely used to treat several diseases, such as arthritis, fever and pain. Their effects are largely attributed to the inhibition of the cyclooxygenase (COX)-mediated synthesis of prostaglandins (PGs) [1]. The selectivity toward the two isoforms of COX-1 and COX-2 varies among different NSAIDs: for example, ibuprofen and naproxen are nonselective COX inhibitors, whereas celecoxib, rofecoxib, diclofenac, and nimesulide are COX-2 selective inhibitors [2]. Recent reports revealed that, in addition to arthritis and pain, cancer and neurodegenerative diseases (e.g., Alzheimer’s disease (AD)) could be treated with some NSAIDs [3,4]. Notably, the brain in AD is characterized by a chronic inflammatory status due to activated glial cells and increased expression of inflammatory cytokines, chemokines and reactive oxygen species (ROS) [5]. Simultaneously, protein aggregates (mainly formed by extracellular deposition of amyloid-β-peptide) andintracellular neurofibrillary agglomerates (formed by hyperphosphorylated tau protein filaments) actively contribute to intensify the neuroinflammation process [6].

To date, the protective effect of the long-term use of NSAIDs against neuroinflammation in AD is confirmed by several epidemiological studies [7]. NSAIDs diminish the risk of AD, delay dementia onset, slowing its progression and reducing the severity of cognitive symptoms [8]. Moreover, NSAIDs are able to alter the conformation of Aβ peptides exerting anti-aggregation activity and induce the expression of amyloid-binding proteins, e.g., transthyretin, that play an important role in sequestering Aβ peptides and preventing their aggregation [9,10].

However, NSAIDs possess gastrointestinal (GI) toxicity due to COX inhibition [11]. In fact, despite countless benefits, chronic use of NSAIDs is limited by their interference with the production of gastrointestinal mucosa, which significantly increases the risk of GI-related side effects, such as dyspepsia, abdominal pain and occasional perforations. On the other hand, COX-2 selective inhibitors, such as rofecoxib, were not effective in patients with mild and moderate AD [12].

**Figure 1:**
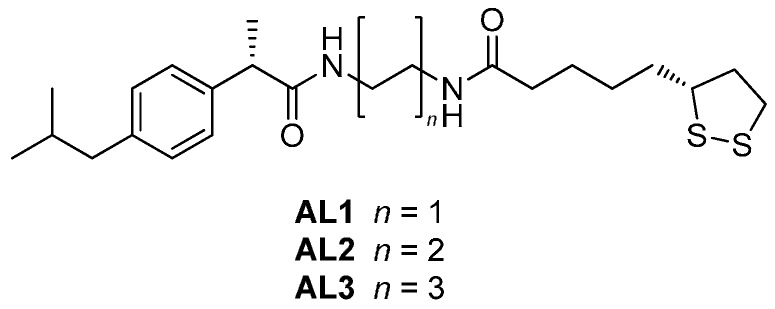
Chemical structures of **AL1**–**3**.

Recently, we synthesized novel lipophilic NSAID conjugates **AL1**–**3** containing (*S*)-ibuprofen (a nonselective Cox inhibitor), chemically linked to a residue of (*R*)-α-lipoic acid (LA), to increase the brain permeability and to reduce the GI side effects of ibuprofen (Figure 1). Furthermore, (*R*)-flurbiprofen derivatives were prepared to improve its permeability into central nervous system (CNS) [13,14,15]. In fact, the brain permeability of NSAIDs is very low since their levels in cerebrospinal fluid (CSF) reach 1%–2% of the plasma levels after administration of therapeutic doses [16]. In vivo studies performed on Aβ-infused AD rats showed that NSAID conjugates **AL1–3** were able to reduce the neuronal damage produced by Aβ(1–40) and, at the same time, limit the neuroinflammation process and the production of ROS [14].

Starting from these data, the aim of this work was to synthesize novel NSAIDs conjugates **AL4**–**9** endowed with neuroprotective properties against neuroinflammation and oxidative stress, which are peculiar to the AD-affected brain. For this purpose, (*S*)-naproxen was selected for the first panel of the compounds **AL4**–**6**. Compared to ibuprofen and other NSAIDs, it possesses marked anti-aggregation properties against Aβ-peptides, since it is able to interfere with the β-sheet conformation of the aggregates, destabilizing them [17], even though it targets Aβ fibrils rather than oligomers [18]. For the second panel of NSAIDs derivatives **AL7**–**9**, we selected (*R*)-flurbiprofen as the anti-inflammatory portion given that studies showed its marked effect on the reduction of Aβ(1–40) and Aβ(1–42) deposition without interfering with γ-secretase activity [19]. An important advantage of using the R-enantiomer of flurbiprofen is the lack of GI side effects, because it has scarce inhibitory activity on COX and exerts its anti-inflammatory action through NF-κB inhibition [20,21]. The presence of LA in both series of NSAID conjugates has the important role of: (1) counteracting ROS that are produced following neuroinflammation process; (2) increasing glutathione levels; and (3) regulating the homeostasis of metals by chelating them [22]. LA was joined to (*S*)-naproxen and (*R*)-flurbiprofen using as chemical linkers alkylene diamine of different lengths; they are considered as naturally “privileged structures” since they possess a wide range of activities and are involved in proliferation processes, neurotransmitter pathways and neuroprotection [23]. Moreover, the temporary block of carboxyl group of the selected NSAIDs, by linkage to the alkylene diamine chain, could guarantee poor gastrointestinal toxicity.

The two series of NSAID conjugates **AL4**–**9**, together with the previously synthesized **AL1**–**3**, were tested on two cellular lines, THP-1 (leukemic monocytes) and U937 (lymphoblast lung from human), to evaluate the neuroprotective activities against toxic stimuli, such as phorbol 12-miristate 13-acetate (PMA), lipopolysaccharide (LPS) and Aβ(25–35). Furthermore, the stability in simulated gastric and intestinal fluids and human plasma was assayed.

## 2. Results and Discussion

### 2.1. Chemistry

**Scheme 1:**
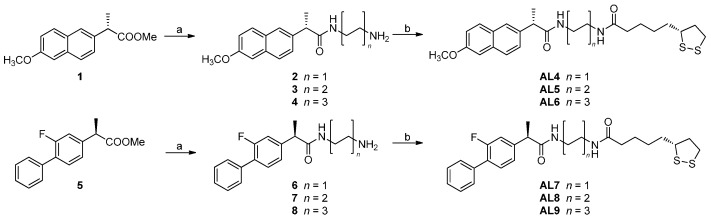
Synthetic routes for the compounds **AL4**–**9**. Reagents and conditions: (**a**) ethylenediamine (for **2** and **6**), butylenediamine (for **3** and **7**) or hexanediamine (for **4** and **8**), 4 h, 120 °C; (**b**) lipoic acid (LA), triethylamine (TEA), hydroxybenzotriazole (HOBt), 1-ethyl-3-(3-dimethylaminopropyl)carbodiimide (EDCI), dry dichloromethane (DCM), 15 h, room temperature.

The synthesis of the compounds **AL4**–**9** was performed as reported in Scheme 1. Following known synthetic pathways, amino-alkylenamides (**2**–**4** and **6**–**8**) were obtained in yields ranging from 36%–80% by the reaction of the NSAID methyl ester (**1** or **5**) with the proper alkylene diamine (ethylene-, butylene- or hexanediamine, respectively) under reflux conditions (120 °C for 4 h) [24]. Final coupling with LA through 1-ethyl-3-(3-dimethylaminopropyl)carbodiimide (EDCI), in the presence of hydroxybenzotriazole (HOBt) and triethylamine (TEA) at room temperature for 15 h, afforded the compounds **AL4**–**9** in good yields (55%–70%) [25].

### 2.2. Stability Studies of **AL4**–**9**

The enzymatic stabilities of **AL1**–**9** were evaluated in simulated fluids (simulated gastric fluid and simulated intestinal fluid, SGF (pH 1.3) and SIF (pH 7.4), respectively), with different concentrations (10 and 40 mg/mL) of enzymes (pepsin and pancreatin) and in human plasma [26]. As reported in Table 1, **AL1**–**9** are quite stable at pH 1.3 in the presence of 10 mg/mL of pepsin (*t*_½_ > 37 h) and pancreatin pH 7.4 (*t*_½_ > 13 h). In the presence of increasing concentrations of the enzymes, the rate of hydrolysis of all compounds was significantly faster at pH 7.4 than 1.3; however, in the presence of pepsin (40 mg/mL) at pH 1.3, the most stable compounds were **AL1** and **AL7** (*t*_½_ > 130 h). The high stability of these compounds at low pH is an advantage for oral administration since they can remain longer in the stomach without undergoing metabolism.

On the other hand, in the presence of 40 mg/mL of pancreatin at pH 7.4, **AL4** was the least stable, showing a half-life of 3.66 h. At the same experimental conditions, **AL1** and **AL7** resulted in being the most stable NSAID derivatives (*t*_½_ > 26 h). In human plasma, as we expected, all derivatives resulted in being less stable due to the well-known presence of plasma esterases (*t*_½_ < 19 h) (Table 2).

Taken together, these results suggest that **AL1** and **AL7** were not susceptible to hydrolysis, both in simulated and physiological environments, hence resulting in being the most stable compounds in all experimentally tested conditions. **AL1** and **AL7**, containing the shortest alkylene diamine chain, displayed better enzymatic stability compared to other derivatives, suggesting that this short chain could be less sensitive to hydrolysis.

### 2.3. Biological Results

Monocytes were chosen as the cell lines to test **AL1**–**9** since these cells are involved in AD etiology by defending the body against pathogens and toxins. Although few data are available about the interaction of monocytes with the brain under physiological conditions, it was seen that in pathological conditions, such as neurodegenerative diseases, a mobilization of pro-inflammatory monocytes directed to the inflamed brain tissues occurs [27]. The rate of infiltration of monocytes increases in response to the inflammatory stimuli present at the cerebral level. Once inside the damaged brain, monocytes evolve to macrophages, which possess a great phagocytic capacity for the Aβ peptide and are responsible for the production of inflammatory mediators [28].

To explore the neuroprotective potential of **AL1**–**9**, all biological experiments were performed on two human monocytic cell lines, U937 and THP-1 cells. Notably, the most important differences between U937 and THP-1 cells are the origin and the maturation stages [29]. U937 cells are pro-monocytic leukemia cells originating from the histiocytic lymphoma of a 37-year-old male, while THP-1 cells are mature monocytic cells derived from the blood of a patient affected by acute leukemia [30].

First, we performed a cell-based assay (MTT assay) to verify if **AL1**–**9** had any effects on cell proliferation or showed cytotoxic effects. The MTT assay is a colorimetric assay for measuring the activity of mitochondrial succinate dehydrogenase, which reduces MTT dyes to formazan, which is a marker of viable cells. Dose-response experiments (with **AL1**–**9** concentrations of 0.1, 1, 10 and 50 μM) were carried out to verify whether, 24 and 48 h after treatment, the compounds had any effect on the cell proliferative capacity (Figures S1 and S2). Results showed that, after 24 and 48 h, **AL1**–**9**, at the concentrations of 0.1 and 1 μM, did not significantly reduce the cellular vitality in THP-1 cells (Figure S1). A marked decrease in cellular vitality was observed when cells were exposed for 24 and 48 h at higher **AL1**–**9** concentrations (10 and 50 μM) (Figure S1). Similar results were obtained treating U937 cells with the same series of compounds. Notably, after 24 and 48 h at the concentrations of 0.1 and 1 μM of **AL1**–**9**, the cellular viability did not significantly decrease; on the other hand, an increase of cellular death was observed augmenting the concentration of **AL1**–**9** up to 50 μM (Figure S2).

Starting from these considerations, we selected **AL7** for further experiments because, differently from other derivatives, it also showed a marked increase of cellular vitality after 48 h in U937 cells at the concentrations of 0.1 and 1 μM. Since **AL7** resulted in the most active NSAID derivative, we also evaluated its stability in fetal bovine serum, which represents the medium used for biological experiments. Our outcomes highlighted that **AL7** is quite stable in the adopted experimental conditions (*t*_½_ > 34 h).

#### 2.3.1. ROS Scavenging Properties of **AL7**

In our experiments, both cell lines, THP-1 and U937, were treated with different stimuli to differentiate them into macrophages [30]. After treatment with phorbol esters, THP-1 and U937 cells differentiate into macrophage-like cells that mimic native monocyte-derived macrophages in several aspects. Notably, PMA represents a good tool to investigate the signal transduction pathways leading to ROS formation in the intracellular compartment, as a consequence of NADPH oxidase activation [31]. PMA can induce activation of the extracellular signal-regulated kinase (ERK) pathway and the c-Jun N-terminal kinase (JNK) pathway. Unlike ERK activation, JNK activation by phorbol esters is somehow cell-specific [32,33].

**Figure 2:**
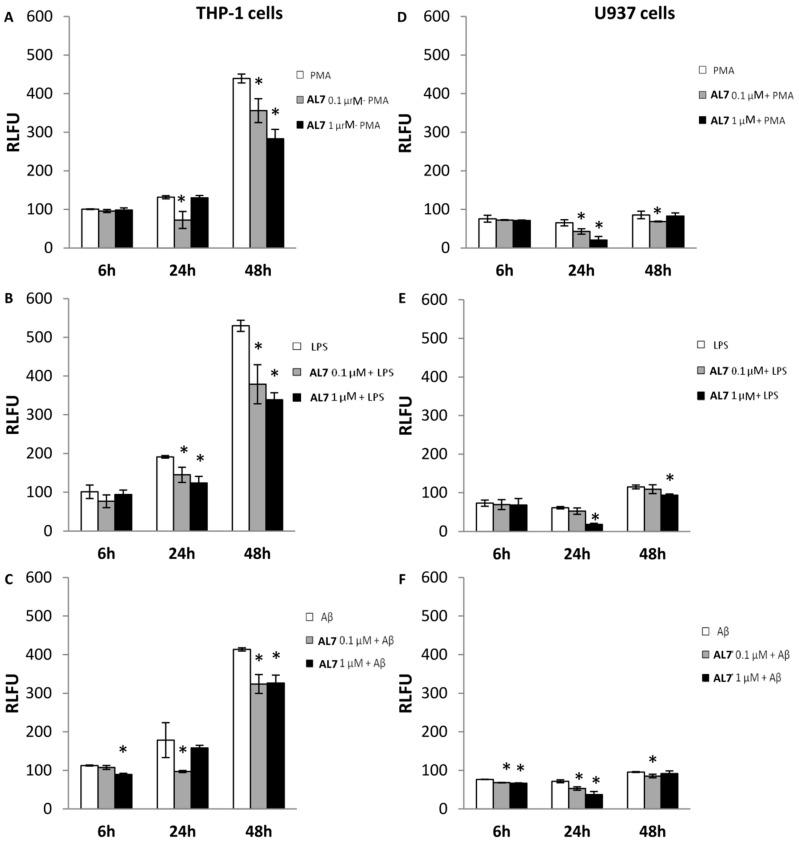
ROS scavenging properties of **AL7** measured by DCFH-DA. Both cell lines were pre-incubated with **AL7** at 0.1 and 1 μM for 6, 24 and 48 h. (**A**,**D**) THP-1 and U937 cells were stimulated with phorbol 12-miristate 13-acetate (PMA) (100 nM); (**B**,**E**) THP-1 and U937 cells were stimulated with LPS (1 μg/μL); (**C**,**F**) THP-1 and U937 cells were stimulated with Aβ(25-35) (10 μM). The means ± SEM derived from three different experiments (each with *n* = 16; * *p* < 0.05, not significative *p* > 0.05).

Literature data report that stimulation of THP-1 and U937 cells with PMA induced ROS release [34]. ROS production was measured using 2′,7′-dichlorofluorescein (DCF), as a fluorescent probe. Thus, in our experiments, we pre-treated both cell lines with **AL7** (0.1 and 1 μM) 6, 24 and 48 h before exposure to PMA (100 nM) to evaluate the capabilities of **AL7** to counteract ROS production (Figure 2A). Results showed that in THP-1 cells the ROS production induced by PMA was significantly reduced by **AL7** after 24 h (0.1 μM) by 44.82% and after 48 h (1 μM) by 35.50%. However, the highest ROS scavenging activity was observed following the treatment of U937 cells with **AL7** (1 μM) 24 h after exposure to PMA (68.34%), suggesting that this cellular line is more sensitive to the protective effect induced by our compound (Figure 2D).

The mechanisms of toxicity of Aβ fragments with different lengths were extensively studied [35]. Among them, the Aβ(25–35) peptide is the shortest fragment of Aβ processed in vivo by brain proteases. Studies showed that this peptide constitutes the biologically-active site of Aβ, retaining the toxicity of the full-length Aβ(1–42) peptide [36]. Its neurotoxic mechanism is correlated with the mitochondrial damage induced after its internalization into cells.

Following the interaction with the mitochondrial inner membrane, Aβ(25–35) interferes with dehydrogenases producing NADH, arousing mitochondrial swelling [37]. In our experiments, both cellular lines were treated with Aβ(25–35), which impairs mitochondrial redox activity and increases the generation of ROS (Figure 2C,F) [38]. Results displayed that Aβ(25–35)-induced ROS production in THP-1 cells was reduced (45.63%) by pretreatment with **AL7** (0.1 μM) after 24 h (Figure 2C); ROS levels were lowered by 48.44% in Aβ(25–35)-stimulated U937 cells after pretreatment with **AL7** 1 μM (Figure 2D–F).

LPS, the component of the outer cell wall of Gram-negative bacteria, is a strong activator for macrophages, both by stimulating ROS, such as H_2_O_2_, superoxides and NO, and inducing the transcription of inflammatory genes [39]. By means of the DA-DCFH fluorescent assay (Figure 2B,E) LPS (1 μg/mL) stimulation of THP-1 and U937 cells rapidly induced ROS through significant H_2_O_2_ production. In contrast, pretreatment with **AL7** (1 μM), containing an antioxidant portion, successfully reduced LPS-induced ROS release in THP-1 cells by 35.31% and 36.01% after 24 and 48 h, respectively (Figure 2B). Furthermore, we observed that pretreatment of cells with **AL7** (1 μM) successfully attenuated LPS-induced H_2_O_2_ elevation in U937 cells 24 h after exposure to LPS (69.49%) (Figure 2E).

Our results evidenced that THP-1 cells are more sensitive to the protective effect of **AL7**; in fact, it exerts its protective activity at low concentration (0.1 μM) in THP-1 cells compared to U937, where a higher concentration is required to counteract ROS (1 μM). Moreover, the presence of LA in the molecule confers antioxidant properties to **AL7** through both direct and indirect mechanisms: LA is able to scavenge hydroxyl radicals and other ROS, HClO, and peroxynitrite by a direct mechanism; on the other hand, it chelates metals (Cu^2+^, Fe^2+^ and Zn^2+^) involved in the onset and progression of AD and induces the expression of cytoprotective genes, such as heme-oxygenase-1 by an indirect mechanism [40]. Moreover, as previously shown [41], the introduction of LA in our molecule could potentiate the antioxidant defense system of cells, since it induces the elevation of glutathione levels, which are reduced in brain affected by AD.

#### 2.3.2. Effect of **AL7** on the Expression of COX-2, IL-1β, and TNF-α in LPS- and Aβ-Stimulated THP-1 Cells

**Figure 3:**
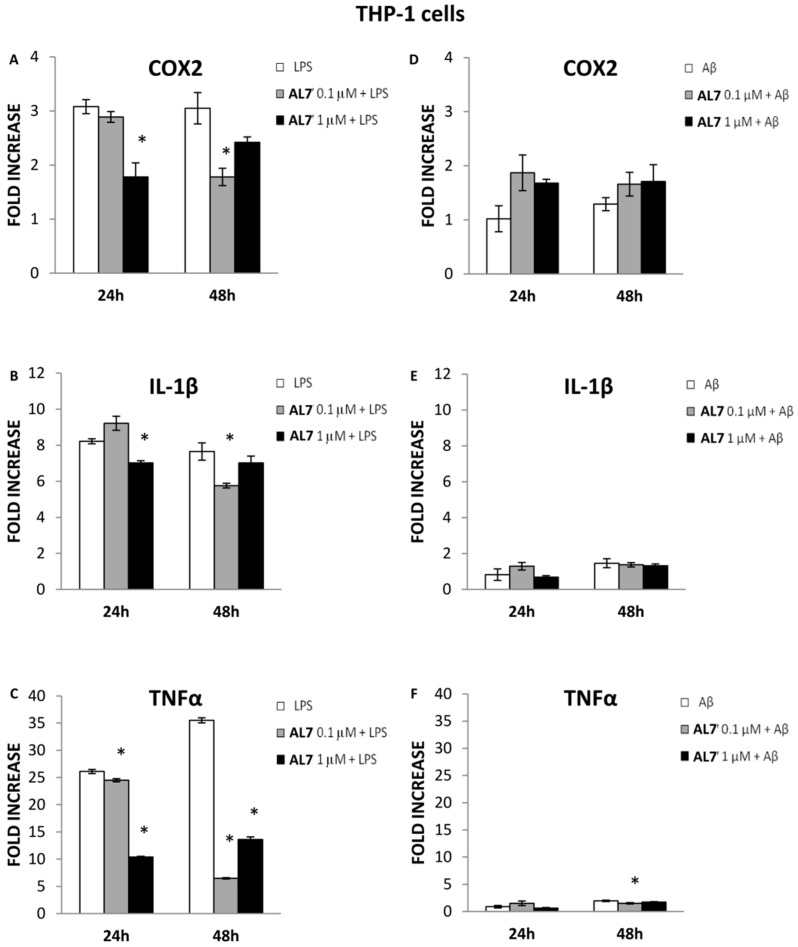
Effect of **AL7** (0.1 and 1 μM) on the expression of COX-2, IL-1β and TNF-α in LPS- and Aβ-stimulated THP-1 cells ((**A**–**C**), (**D**–**F**), respectively). The means ± SEM derived from three different experiments (each with *n* = 16; * *p* < 0.05, not significative *p* > 0.05).

The stimulation of THP-1 macrophages with LPS induces an increased expression of inflammatory cytokines (IL-1, IL-6 and IL-8), inflammatory enzymes (COX-2 and iNOS) and transcription factors (TNF-α) [42]. Figure 3 reports the mRNA expression levels of the pro-inflammatory cytokine genes IL-1 and TNF-α and the inflammation-related enzyme gene COX-2 determined by RT-PCR. Results showed that the exposure of THP-1 cells to LPS, after 24 and 48 h, induced marked expression of all of the above genes reported. **AL7** strongly reduced TNF-α (60.15%) and COX-2 (42.21%) at 24 h and a higher concentration (1 μM), while a mild activity was observed for IL-1 (14.60%). A lower concentration (0.1 μM) of **AL7** significantly decreased the LPS-induced expression of TNF-α (81.77%) and COX-2 (41.64%), although requiring more time (48 h).

Moreover, THP-1 cells were treated with Aβ(25–35), a short peptide endowed with the same toxicity of Aβ(1–40) and Aβ(1–42). This stimulus is a strong inducer of the release of pro-inflammatory cytokines and enzymes [43]. After 24 h, **AL7** at 1 μM reduced IL-1 (16.59%) and TNF-α (28.56%) expression, while after 48 h and at higher concentration, it significantly lowered TNF-α gene expression (23.08%). On the other hand, **AL7** induced a marked increase in COX-2 expression.

All of these data demonstrated that **AL7** possesses direct anti-inflammatory properties in LPS-stimulated THP-1 cells. However, a minor effect was observed when Aβ(25–35)-stimulated cells were treated with **AL7**, suggesting that THP-1 cells respond in a different way to both toxic stimuli.

Aβ(25–35)-induced neurotoxicity involves augmented COX-2 enzymes that produce free radicals responsible for oxidative stress, which potentiates Aβ neurotoxicity, thus triggering a vicious circle. The raised expression of COX-2 in Aβ(25–35)-treated cells after treatment with **AL7** could be due to the incapability of **AL7** to solve the inflammation, leading to a persistent activation of the inflammatory cascade (Figure 3D). Probably, the presence of (*R*)-flurbiprofen in the molecule justifies the scarce COX-2 inhibitory activity, since it is a weak COX inhibitor. In fact, as reported in the literature, (*R*)-flurbiprofen exerts its anti-inflammatory activity by inhibiting the NF-κB factor, not COX activity [20].

#### 2.3.3. Effect of **AL7** on the Expression of COX-2, IL-1β, and TNF-α in LPS- and Aβ-Stimulated U937 Cells

**Figure 4:**
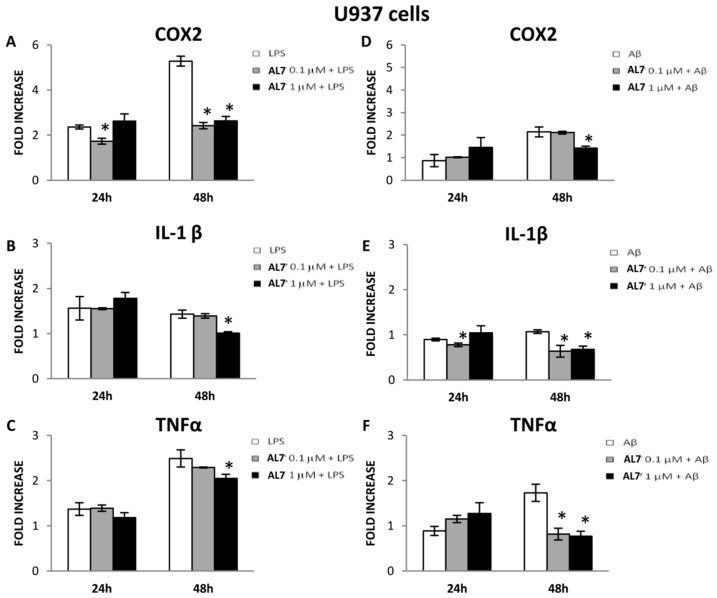
Effect of **AL7** (0.1 and 1 μM) on the expression of COX-2, IL-1β and TNF-α in LPS- and Aβ-stimulated U937 cells ((**A**–**C**), (**D**–**F**), respectively). The means ± SEM derived from three different experiments (each with *n* = 16; * *p* < 0.05, not significative *p*> 0.05).

U937 cells were stimulated with LPS and Aβ(25–35) at 24 and 48 h (Figure 4). After 24 h, following both toxic stimuli, **AL7** pretreatment was not able to exert any anti-inflammatory effect, probably due to the different nature of U937 compared to THP-1 cells. The compound requires a higher time of exposure to decrease the enzymes and genes involved in the inflammatory pathways.

After 48 h, **AL7** displayed protective effects at the concentration of 1 μM against LPS stimulus, since it significantly lowered COX-2 expression by 50.19%, IL-1β expression by 29.37% and TNF-α by 17.67% (Figure 4A–C). The protective effect of **AL7** (1 μM) was also observed after 48 h of treatment with Aβ(25–35): it significantly lowered COX-2 levels (33.18%), IL-1β (36.54%) and TNF-α (55.49%) (Figure 4D–F). At a lower concentration and after 48 h, IL-1β and TNF-α expressions were also significantly reduced (40.65% and 52.83%, respectively) in Aβ(25–35)-stimulated U937 cells (Figure 4E,F).

Our current findings suggest that the anti-inflammatory effect of **AL7** is more pronounced at higher concentrations and after 48 h in the U937 cell line compared to the THP-1 cells.

## 3. Materials and Methods

All reagents and solvents, unless differently stated, were used as received from Sigma Aldrich (St. Louis, MO, USA). Chromatography was performed on silica gel (Merck 60, 70–230 mesh); homogeneity was confirmed by TLC on silica gel Merck 60 F_254_ (Sigma Aldrich, St. Louis, MO, USA). ^1^H- and ^13^C-NMR spectra were recorded on a Varian VXR-300 spectrometer (Varian Inc., Palo Alto, CA, USA). The LC-MS/MS system used consisted of an LCQ ion trap mass spectrometer (Thermo Finnigan, San Jose, CA, USA) equipped with an electrospray ionization (ESI) source. HR-MS spectra were recorded using Q Exactive™ Hybrid Quadrupole-Orbitrap™ Mass Spectrometer (Thermo Fischer Scientific, Waltham, MA, USA) coupled with HPLC Dionex series Ultimate 3000 (Thermo Fischer Scientific, Waltham, MA, USA) and a Phenomenex Luna 5µ C18 150 mm × 2 mm (P/N° 00F-4041-B0) column. Mass resolution was set at 140,000, and the scan range was from 250–750 *m*/*z* (Table S1).

### 3.1. Chemistry

#### 3.1.1. General Method for Coupling with Alkylendiamine

A mixture of NSAID methyl ester (**1** or **5**) (3.1 mmol) and the alkylendiamine (ethylenediamine, butylenediamine, or hexanediamine) (122 mmol) was refluxed for 4 h at 120 °C under stirring. Subsequently, the reaction was quenched with water, acidified with HCl 1 N and washed with CHCl_3_ to remove the unreacted ester; then, the aqueous phase was treated with NaHCO_3_ and extracted with CHCl_3_. The organic layer was dried over anhydrous Na_2_SO_4_ prior to evaporation, providing **2**–**4** and **6**–**8**, respectively, which were used without further purifications.

(*S*)-*N*-(2-Aminobutyl)-2-(6-methoxynaphthalen-2-yl)propanamide (**3**). Yield: 36%; R*_f_* = 0.06, CHCl_3_/MeOH (95:5); ^1^H-NMR (300.2 MHz, CDCl_3_) δ: 1.30 (2H, br, s), 1.32–1.44 (4H, m), 1.58 (3H, d, *J* = 6.9 Hz), 2.58 (2H, t, *J* = 6.9 Hz), 3.17 (2H, q, *J* = 6.3 Hz), 3.67 (1H, q, *J* = 7.2 Hz), 3.91 (3H, s), 5.79 (1H, br, s), 7.11–7.72 (6H, m); ^13^C-NMR (75.49 MHz, CDCl_3_) δ: 18.7, 27.2, 30.9, 39.7, 41.8, 47.3, 55.6, 105.8, 119.4, 126.4, 126.6, 127.7, 129.2, 129.4, 133.9, 136.9, 157.9, 174.6; MS (ESI) *m*/*z* 301.19 [M + H]^+^.

(*S*)-*N*-(6-Aminohexyl)-2-(6-methoxynaphthalen-2-yl)propanamide (**4**). Yield: 80%; R*_f_* = 0.11, CHCl_3_/MeOH (95:5); ^1^H-NMR (300.2 MHz, CDCl_3_) δ: 1.18 (2H, br, s), 1.15–1.51 (8H, m), 1.57 (3H, d, *J* = 7.2 Hz), 2.54–2.66 (2H, m), 3.14 (2H, q, *J* = 6.6 Hz), 3.65 (1H, q, *J* = 6.9 Hz), 3.89 (3H, s), 5.46 (1H, br, s), 7.10–7.71 (6H, m); ^13^C-NMR (75.49 MHz, CDCl_3_) δ: 18.7, 26.6, 26.8, 29.7, 33.7, 39.7, 42.2, 47.3, 55.6, 105.8, 119.4, 126.3, 126.6, 127.7, 129.2, 129.4, 133.9, 136.9, 157.9, 174.5; MS (ESI) *m*/*z* 329.22 [M + H]^+^.

(*R*)-*N*-(2-Aminoethyl)-2-(2-fluoro-[1,1′-biphenyl]-4-yl)propanamide (**6**). Yield: 67%; R*_f_* = 0.20, CHCl_3_/MeOH (7:3); ^1^H-NMR (300.2 MHz, CDCl_3_) δ: 1.30 (2H, br, s), 1.53 (3H, d, *J*= 8.4 Hz), 2.73–2.80 (2H, m), 3.23–3.29 (2H, m), 3.57–3.59 (1H, m), 6.08 (1H, br, s), 7.11–7.54 (8H, m); ^13^C-NMR (75.49 MHz, CDCl_3_) δ: 18.9, 41.5, 42.4, 46.9, 115.3 (d, 2*J*_C,F_ = 23.5 Hz), 123.8(d, 4*J*_C,F_ = 3.4 Hz), 128.0 (d, 2*J*_C,F_ = 13.2 Hz), 128.7 (× 2), 129.1 (× 2), 131.2 (d, 3*J*_C,F_ = 4.0 Hz), 135.6, 143.2 (d, 3*J*_C,F_ = 7.42 Hz), 160.0 (d, 1*J*_C,F_ = 248.5 Hz), 174.0; MS (ESI) *m*/*z* 287.15 [M + H]^+^.

(*R*)-*N*-(6-Aminohexyl)-2-(2-fluoro-[1,1′-biphenyl]-4-yl)propanamide (**8**). Yield: 73%; R*_f_* = 0.14, CHCl_3_/MeOH (6:4); ^1^H-NMR (300.2 MHz, CDCl_3_) δ: 1.22 (2H, br, s), 1.32–1.47 (8H, m), 1.53 (3H, d, *J* = 7.2 Hz), 2.61–2.66 (2H, t, *J* = 6.9 Hz), 3.21 (2H, q, *J* = 6 Hz), 3.55 (1H, q, *J* = 7.2 Hz), 5.44 (1H, br, s), 7.09–7.55 (8H, m); ^13^C-NMR (75.49 MHz, CDCl_3_) δ: 18.8, 26.7, 26.8, 29.7, 33.8, 39.9, 42.3, 47.0, 115.5 (d, 2*J*_C,F_ = 23.5 Hz), 123.8 (d, 4*J*_C,F_ = 3.5 Hz), 128.0 (d, 2*J*_C,F_ = 13.3 Hz), 128.7 (× 2), 129.1 (× 2), 131.2 (d, 3*J*_C,F_ = 4.0 Hz), 135.6, 143.1 (d, 3*J*_C,F_ = 7.42 Hz), 160.0 (d, 1*J*_C,F_ = 248.7 Hz), 173.6; MS (ESI) *m*/*z* 343.21 [M + H]^+^.

#### 3.1.2. General Method for Coupling with LA

A stirred solution of LA (0.99 mmol) in dry dichloromethane (DCM) (4.1 mL) was added with the NSAID-amino-alkylenamide (**2**–**4** or **6**–**8**) (0.9 mmol), TEA (21.16 mmol), HOBt (1.29 mmol) and EDCI (2.57 mmol). The mixture was allowed to react overnight at room temperature [44,45]. After quenching with water, the product was extracted with DCM; the organic layer was dried over anhydrous MgSO_4_; and the solvent was removed under vacuum. The crude was chromatographed with CHCl_3_/MeOH (95:5) to afford the compounds **AL4**–**9**. All final compounds were fully characterized by NMR spectroscopy and HR-MS (Figures S3–S17).

5-((*R*)-1,2-Dithiolan-3-yl)-*N*-(2-((*S*)-2-(6-methoxynaphthalen-2-yl)propanamido)ethyl)pentanamide (**AL4**). Yield: 66%; R*_f_* = 0.54, CHCl_3_/MeOH (95:5); ^1^H-NMR (300.2 MHz, CDCl_3_) δ: 1.23–3.13 (15H, m), 3.28–3.33 (4H, m), 3.46–3.55 (1H, m), 3.65–3.71 (1H, m), 3.91 (3H, s), 6.04 (1H, br, s), 6.06 (1H, br, s), 7.11–7.73 (6H, m); ^13^C-NMR (75.48 MHz, CDCl_3_) δ: 18.6, 25.4, 29.1, 34.8, 36.4, 38.7, 40.2, 40.4, 40.5, 47.2, 55.6, 56.7, 105.8, 119.5, 126.3, 127.8, 129.2, 129.5, 129.5, 134.0, 136.5, 158.0, 173.9, 176.1; HR-MS (ESI) *m*/*z* 461.1922 [M + H]^+^.

5-((*R*)-1,2-Dithiolan-3-yl)-*N*-(4-((*S*)-2-(6-methoxynaphthalen-2-yl)propanamido)butyl)pentanamide (**AL5**). Yield: 61%; R*_f_* = 0.57, CHCl_3_/MeOH (95:5); ^1^H-NMR (300.2 MHz, CDCl_3_) δ: 1.39–1.45 (8H, m), 1.59 (3H, d, *J* = 7.2 Hz), 1.62–1.71 (4H, m), 1.86–2.46 (4H, m), 3.09–3.21 (4H, m), 3.54–3.57 (1H, m), 3.65–3.71 (1H, m), 3.92 (3H, s), 5.61–5.64 (2H, m), 7.13–7.73 (6H, m); ^13^C-NMR (75.48 MHz, CDCl_3_) δ: 18.7, 25.6, 26.8, 27.2, 29.1, 34.8, 36.7, 38.7, 39.2, 39.3, 40.5, 47.3, 55.6, 56.70, 105.8, 119.5, 126.4, 126.5, 127.8, 129.2, 129.5, 134.0, 136.8, 158.0, 173.0, 174.8; HR-MS (ESI) *m*/*z* 489.2236 [M + H]^+^.

5-((*R*)-1,2-Dithiolan-3-yl)-*N*-(6-((*S*)-2-(6-methoxynaphthalen-2-yl)propanamido)hexyl)pentanamide (**AL6**). Yield: 70%; R*_f_* = 0.21, CHCl_3_/MeOH (99:1); ^1^H-NMR (300.2 MHz, CDCl_3_) δ: 1.17–1.48 (10H, m), 1.58 (3H, d, *J* = 6.9 Hz), 1.62–1.73 (4H, m), 1.83–1.94 (1H, m), 2.13–2.20 (2H, m), 2.38–2.49 (1H, m), 3.05–3.25 (6H, m), 3.50–3.59 (1H, m), 3.68 (1H, q, *J* = 7.2 Hz), 3.91 (3H, s), 5.48 (1H, br, s), 5.61 (1H, br, s), 7.12–7.73 (6H, m); ^13^C-NMR (75.48 MHz, CDCl_3_) δ: 18.7, 25.7, 26.0, 26.0, 29.2, 29.6, 29.8, 34.9, 36.8, 38.7, 39.1, 39.2, 40.5, 47.3, 55.6, 56.7, 105.8, 119.4, 126.3, 126.6, 127.8, 129.2, 129.5, 133.9, 136.8, 157.9, 173.0, 174.6; HR-MS (ESI) *m*/*z* 517.2549 [M + H]^+^.

5-((*R*)-1,2-Dithiolan-3-yl)-*N*-(2-((*R*)-2-(2-fluoro-[1,1′-biphenyl]-4-yl)propanamido)ethyl)pentanamide (**AL7**). Yield: 56%; R*_f_* = 0.17, benzene/EtOAc/MeOH (3:7:0.5); ^1^H-NMR (300.2 MHz, CDCl_3_) δ: 1.38–1.42 (2H, m), 1.53 (3H, d, *J* = 6.0 Hz), 1.56–1.65 (4H, m), 1.80–1.90 (1H, m), 2.11 (2H, t, *J* = 9.0 Hz), 2.35–2.45 (1H, m), 3.08–3.16 (2H, m), 3.34 (4H, br, s), 3.51–3.59 (2H, m), 6.04 (1H, br, s), 6.38 (1H, br, s), 7.10–7.54 (8H, m); ^13^C-NMR (75.48 MHz, CDCl_3_) δ: 18.6, 25.6, 29.1, 34.8, 36.4, 38.7, 40.0, 40.4, 40.6, 46.6, 56.6, 115.4 (d, 2*J*_C,F_ = 23.5 Hz), 123.8 (d, 4*J*_C,F_ = 3.2 Hz), 128.0 (d, 3*J*_C,F_ = 7.2 Hz), 128.7 (× 2), 129.1 (× 2), 131.1 (d, 3*J*_C,F_ = 4.0 Hz), 135.6, 142.9 (d, 3*J*_C,F_ = 7.65 Hz), 159.9 (d, 1*J*_C,F_ = 248.8 Hz), 174.5, 175.2; HR-MS (ESI) *m*/*z* 475.1880 [M + H]^+^.

5-((*R*)-1,2-Dithiolan-3-yl)-*N*-(4-((*R*)-2-(2-fluoro-[1,1′-biphenyl]-4-yl)propanamido)butyl)pentanamide (**AL8**). Yield: 55%; R*_f_* = 0.26, benzene/EtOAc/MeOH (3:7:0.5); ^1^H-NMR (300.2 MHz, CDCl_3_) δ: 1.39–1.49 (6H, m), 1.53 (3H, d, *J* = 6.0 Hz), 1.57–1.75 (4H, m), 1.83–1.94 (1H, m), 2.16 (2H, t, *J* = 6.0 Hz), 2.38–2.49 (1H, m), 3.05–3.20 (2H, m), 3.20–3.28 (4H, m), 3.50–3.62 (2H, m), 5.76 (1H, t, *J* = 4.0 Hz), 5.93 (1H, t, *J* = 4.0 Hz), 7.11-7.55 (8H, m); ^13^C-NMR (75.48 MHz, CDCl_3_) δ: 18.8, 25.7, 26.9, 27.2, 29.1, 34.8, 36.7, 38.7, 39.1, 39.5, 40.5, 46.8, 56.7, 115.5 (d, 2*J*_C,F_ = 23.5 Hz), 123.8 (d, 4*J*_C,F_ = 3.5 Hz), 128.0 (d, 2*J*_C,F_ = 7.7 Hz), 128.7 (× 2), 129.1 (× 2), 131.2 (d, 3*J*_C,F_ = 4.0 Hz), 135.6, 143.1 (d, 3*J*_C,F_ = 7.42 Hz), 160.0 (d, 1*J*_C,F_ = 248.5 Hz), 173.3, 174.0; HR-MS (ESI) *m*/*z* 503.2193 [M + H]^+^.

5-((*R*)-1,2-Dithiolan-3-yl)-*N*-(6-((*R*)-2-(2-fluoro-[1,1′-biphenyl]-4-yl)propanamido)hexyl)pentanamide (**AL9**). Yield: 55%; R*_f_* = 0.35, CHCl_3_/MeOH (6:4); ^1^H-NMR (300.2 MHz, CDCl_3_) δ: 1.22–1.27 (4H, m), 1.40–1.46 (6H, m), 1.53 (3H, d, *J* = 6.3 Hz), 1.61–1.69 (4H, m), 1.82–1.91 (1H, m), 2.12 (2H, t, *J* = 7.5 Hz), 2.40–2.46 (1H, m), 3.08–3.24 (6H, m), 3.51–3.60 (2H, m), 5.69 (1H, t, *J* = 4.8 Hz), 5.78 (1H, t, *J* = 4.8 Hz), 7.11–7.54 (8H, m); ^13^C-NMR (75.48 MHz, CDCl_3_) δ: 18.8, 25.7, 25.9, 26.0, 29.2, 29.5, 29.7, 34.9, 36.7, 38.7, 39.0, 39.3, 40.5, 46.8, 56.7, 115.4 (d, 2*J*_C,F_ = 23.5 Hz), 123.8 (d, 4*J*_C,F_ = 3.5 Hz), 128.0 (d, 2*J*_C,F_ = 6.0 Hz), 128.7 (× 2), 129.1 (× 2), 131.2 (d, 3*J*_C,F_ = 4.0 Hz), 135.6, 143.2 (d, 3*J*_C,F_ = 7.42 Hz), 160.0 (d, 1*J*_C,F_ = 248.5 Hz), 173.1, 173.8; HR-MS (ESI) *m*/*z* 531.2504 [M + H]^+^.

### 3.2. Pharmacokinetic Studies

#### 3.2.1. HPLC-UV Assays

Analytical HPLC measurements were run on a Waters 600 HPLC pump (Waters Corporation, Milford, MA, USA), equipped with a Waters 2996 photodiode array detector. The column was a Luna C18 column (250 mm × 4.6 mm, 5 μm). The mobile phase was a mixture of H_2_O/ACN flushing at a rate of 0.8 mL/min under isocratic conditions.

#### 3.2.2. Drug Stability in Simulated Gastric and Intestinal Fluids (SGF and SIF)

The enzymatic stability was evaluated in hydrochloric buffer with pepsin and in phosphate buffer containing pancreatin at two different enzymatic concentrations (10 and 40 mg/mL). Two hundred microliters of **AL1**–**9** stock solution were added to a preincubated buffer solution (1 mL), at 37 °C and 650 rpm, and shaken at 37 °C and 650 rpm. Samples of 100 μL were withdrawn at various times and deproteinized by mixing with 100 μL of ice-cold acetonitrile containing 0.5% *v*/*v* of formic acid. The samples were vortexed and centrifuged at 2 °C and 10,000 rpm for 10 min. The drug content in the supernatant was analyzed by HPLC [46].

#### 3.2.3. Drug Stability in Human Plasma

To assess the enzymatic hydrolysis, the drug stock solution was added with a pre-heated (37 °C) plasma fraction, previously diluted with 0.02 M phosphate buffer (pH 7.4) to give a final volume of 1 mL (80% plasma). Samples of 100 μL were taken at various times, and 200 μL of 0.01 M HCl in methanol were added to stop the enzymatic activity. After centrifugation for 5 min at 5000× *g*, the supernatant was analyzed by HPLC [47]. Plasma was purchased from 3H Biomedical (Uppsala, Sweden).

#### 3.2.4. Stability Studies in Fetal Bovine Serum

In vitro enzymatic hydrolysis of **AL7** was studied in fetal bovine serum at 37 °C. **AL7** stock solution was added to preincubated bovine serum with 0.02 M phosphate buffer (pH 7.4). Aliquots of 100 μL were removed at pre-determined time points and deproteinized with 200 μL of 0.01 M HCl in methanol. The mixture was centrifuged at 3000× *g* for 10 min. The supernatant was then removed and analyzed by HPLC [48].

### 3.3. Biological Assays

#### 3.3.1. Cell Lines

The human monocytic leukemia cell line THP-1 and the pro-monocytic human myeloid leukemia cell line U937 were purchased from the American Type Culture Collection (Rockville, MD, USA) and maintained in RPMI 1640 (Sigma Aldrich, St. Louis, MO, USA) containing 10% heat-inactivated fetal bovine serum (Sigma Aldrich), 2 mM l-glutamine and 10 mM HEPES, at 37 °C in a humidified atmosphere of 5% CO_2_.

#### 3.3.2. MTT Assay

THP-1 and U937 cells were seeded on 96-well plates at a density equal to 1 × 10^5^ in 100 μL of growth media and preincubated with **AL7** at the concentrations of 0.1 and 1 μM for 1 h. After this preincubation, Aβ(25–35) (10 μM), LPS (1 μg/μL) and PMA (100 nM) were added to cells and maintained in culture for 6, 24, and 48 h. At the end of this treatment period, the growth media were removed and replaced by PBS containing 10 μM 3-(4,5-dimethylthiazol-2-yl)-3,5-diphenylformazan (MTT), and cells were incubated for 2 h at 37 °C. After the incubation period, the MTT medium was removed and replaced with an equal volume of DMSO to break the cell membranes and solubilize the intracellular purple crystals. The absorbance of the resulting purple solution was measured at 550 nm with the GloMax-Multi^+^ Detection System (Promega, Madison, WI, USA). All assays were performed in triplicate.

#### 3.3.3. ROS Assay

THP-1 and U937 cells were preincubated with **AL7** at the concentrations of 0.1 and 1 μM for 6, 24, and 48 h. After this period, the growth media were removed, and the carboxy-2′,7′-dichloro-dihydro-fluorescein diacetate (DCFH-DA) probe was added to cells at the concentration of 20 μM in PBS and incubated for 30 min at 37 °C. Afterwards, the medium was removed, and the cells, washed with PBS, were seeded on 96 black well plates at a density equal to 1 × 10^5^ in 100 μL of PBS. Cell fluorescence was determined with and without the inducer Aβ(25–35) (10 μM) in control cells and in **AL7**-treated cells. To create the positive control, oxidative activity was stimulated by LPS (1 μg/μL) and PMA (100 nM), both in control and **AL7**-treated cells. The fluorescence was immediately read after the addition of the inducer with the GloMax-Multi^+^ Detection System (Promega, Madison, WI, USA) using Excitation 490 nm, Emission 510–570 nm. All assays were performed in triplicate.

#### 3.3.4. RNA Extraction, Reverse Transcription (RT-PCR) and Real-Time PCR

Total RNA was extracted from THP-1 and U937 cells, after the culture, using TRIzol reagent (Invitrogen, Life Technologies, Paisley, United Kingdom). The RNA concentration was determined by measuring the samples’ absorbance (λ = 260 nm) using the NanoDrop 2000 UV-Vis Spectrophotometer (Thermo Scientific, Waltham, MA, USA); its purity was assessed by the absorbance ratios λ 260/280 nm and λ 260/230 nm. For each sample, 1 μg of RNA was reverse transcribed into complementary DNA (cDNA) using the Quanti Tect Reverse Transcription Kit (Qiagen, Venlo, Limburg, The Netherlands). SYBRGreen-based Real-Time PCR, with a melting curve analysis, was performed with GoTaq^®^ qPCR Master Mix (Promega, Madison, WI, USA) using the cDNA and specific primer pairs, to evaluate the gene expression of COX-2 (forward, 5′-GACAGTCCACCAACTTACAATG-3′ and reverse, 5’-GGCAATCATCAGGCACAGG-3′), IL-1β (forward, 5′-TGAGGATGACTTGTTCTTTGAAG-3′ and reverse, 5′-GTGGTGGTCGGAGATTCG-3′) and TNF-α (forward, 5′-CCTTCCTGATCGTGGCAG-3′ and reverse, 5′-GCTTGAGGGTTTGCTACAAC-3′). All PCR reactions were performed in triplicate with the thermal cycler Mastercycler ep (Eppendorf, Hamburg, Germany) with the following conditions: initially 2 min incubation at 95 °C followed by 40 cycles consisting in 30 s at 95 °C, then 1 min at 60 °C and 30 s at 68 °C. The analysis of the melting curve was performed in the temperature range of 60–95 °C at the end of each run.

The quantitative reverse-transcription polymerase chain reaction (qRT-PCR) was performed in an Eppendorf Mastercycler EP Realplex (Eppendorf, Hamburg, Germany) on individual genes. Each gene-specific primer was used, and 18S was used as the internal control. The relative expression of each gene was normalized by 18S using the Δ*C*_t_ method, where Δ*C*_t_ = *C*_t_ (COX-2, IL-1β and TNF-α) − *C*_t_ (18S) [49]. Predicted cycle threshold values were directly exported into Excel worksheets for analysis. Relative changes in gene expression were determined by the 2^−ΔΔ*C*t^ method, were ΔΔ*C*_t_ = Δ*C*_t_ (experimental sample) −Δ*C*_t_ (calibrator) and reported as the difference (*n*-fold) relative to the value for a calibrator cDNA (control = 1) prepared in parallel with the experimental DNAs [50]. The fold increase with respect to constitutive cells (mean ± SD) is representative of a total of three replicates performed for each experiment (*n* = 9).

## 4. Conclusions

The development of novel strategies to manage AD is urgently needed since the present pharmacological therapies are not able to stop the progression of the pathology, but only to meliorate the symptoms.

The recent interest in non-steroidal anti-inflammatory drugs for the treatment of Alzheimer’s disease is based on their potential anti-amyloid properties. The combination of traditional anti-inflammatory drugs and antioxidant molecules could be an important tool to discover novel multi-target compounds able to simultaneously counteract neuroinflammation and oxidative stress that constitute the hallmarks of such pathology. It is important to understand how these novel molecules interact with the multiple targets involved in the disease and which are the pathological pathways that they are able to modulate or inhibit. In this context, deepened studies performed on rats affected by AD could elucidate the real therapeutical potential of our new molecules.

## Figures and Tables

**Table 1:** Enzymatic stability studies of **AL1**–**9** ^a^.

Experimental Conditions	Kinetic Parameters	AL1	AL2	AL3	AL4	AL5	AL6	AL7	AL8	AL9
pH 1.3; Pepsin 10 mg/mL	*t*_½_ (h)	187.56 ± 1.24	28.84 ± 0.90	73.52 ± 1.33	144.82 ± 9.94	128.38 ± 3.08	37.73 ± 0.77	138.06 ± 9.24	76.16 ± 0.66	86.61 ± 0.77
	*k*_obs_ (h^−^^1^)	(3.70 ± 0.02) × 10^−3^	(24.16 ± 2.43) × 10^−3^	(9.43 ± 0.17) × 10^−3^	(4.80 ± 0.33) × 10^−3^	(5.40 ± 0.13) × 10^−3^	(18.37 ± 0.38) × 10^−3^	(5.03 ± 0.34) × 10^−3^	(9.10 ± 0.31) × 10^−3^	(8.0 ± 0.06) × 10^−3^
pH 1.3; Pepsin 40 mg/mL	*t*_½_ (h)	157.10 ± 4.55	22.07 ± 2.00	33.61 ± 1.68	29.68 ± 1.61	96.58 ± 7.13	34.35 ± 0.10	130.49 ± 8.22	33.05 ± 0.24	53.20 ± 0.42
	*k*_obs_ (h^−^^1^)	(4.41 ± 0.13) × 10^−3^	(31.54 ± 2.86) × 10^−3^	(20.64 ± 1.03) × 10^−3^	(23.39 ± 1.27) × 10^−3^	(7.20 ± 0.53) × 10^−3^	(20.18 ± 0.06) × 10^−3^	(5.32 ± 0.34) × 10^−3^	(20.97 ± 1.34) × 10^−3^	(13.03 ± 0.93) × 10^−3^
pH 7.4; Pancreatin 10 mg/mL	*t*_½_ (h)	31.93 ± 0.93	17.44 ± 0.15	49.96 ± 0.37	13.73 ± 1.02	67.40 ± 0.54	20.84 ± 0.13	40.26 ± 5.50	34.21 ± 0.22	27.72 ± 0.12
	*k*_obs_ (h^−^^1^)	(21.26 ± 0.64) × 10^−3^	(39.74 ± 0.32) × 10^−3^	(13.87 ± 0.73) × 10^−3^	(50.62 ± 3.77) × 10^−3^	(10.28 ± 0.19) × 10^−3^	(33.25 ± 0.13) × 10^−3^	(17.38 ± 2.38) × 10^−3^	(20.26 ± 0.33) × 10^−3^	(25.0 ± 0.46) × 10^−3^
pH 7.4; Pancreatin 40 mg/mL	*t*_½_ (h)	26.04 ± 1.46	11.18 ± 0.07	15.54 ± 0.13	3.66 ± 0.57	24.52 ± 0.17	7.35 ± 0.05	33.88 ± 0.62	16.50 ± 0.12	19.58 ± 0.13
	*k*_obs_ (h^−^^1^)	(26.65 ± 1.49) × 10^−3^	(61.99 ± 1.78) × 10^−3^	(44.59 ± 0.70) × 10^−3^	(191.76 ± 30.05) × 10^−3^	(28.26 ± 0.37) × 10^−3^	(94.23 ± 0.70) × 10^−3^	(20.46 ± 0.38) × 10^−3^	(42.0 ± 0.02) × 10^−3^	(35.39 ± 0.52) × 10^−3^

^a^ Values are the means of three experiments ± SD.

**Table 2:** Plasma stability studies of **AL1**–**9** ^a^.

Experimental Conditions	Kinetic Parameters	AL1 ^b^	AL2 ^b^	AL3 ^b^	AL4	AL5	AL6	AL7	AL8	AL9
Human Plasma	*t*_½_ (h)	3.00 ± 0.13	2.02 ± 0.06	1.92 ± 0.07	15.95 ± 0.09	12.10 ± 0.11	13.00 ± 0.17	19.33 ± 0.75	13.97 ± 0.11	16.11 ± 0.15
	*k*_obs_ (h^−^^1^)	(230 ± 10.2) × 10^−3^	(340 ± 8.24) × 10^−3^	(360 ± 13.18) × 10^−^^3^	(43.45 ± 0.86) × 10^−3^	(57.27 ± 0.19) × 10^−3^	(53.33 ± 0.71) × 10^−3^	(35.89 ± 1.39) × 10^−3^	(49.61 ± 0.32) × 10^−3^	(43.02 ± 0.06) × 10^−3^

^a^ Values are the means of three experiments ± SD; ^b^ data previously determined [12].

## Supplementary Materials

Supplementary materials can be found at http://www.mdpi.com/1422-0067/17/7/1035/s1.
