# Supplementary material for: Novel NSAID-Derived Drugs for the Potential Treatment of Alzheimer’s Disease
Source: Int J Mol Sci. 2016 Jun 30;17(7):1035. doi: 10.3390/ijms17071035 (PMC4964411; doi:10.3390/ijms17071035)
Supplement: Supplementary file 1 [file ijms-17-01035-s001.pdf]

Supplementary Materials: Novel NSAID-Derived Drugs for the Potential Treatment of Alzheimer's Disease

Ivana Cacciatore, Lisa Marinelli, Erika Fornasari, Laura S. Cerasa, Piera Eusepi, Hasan Türkez, Cristina Pomilio, Marcella Reale, Chiara D'Angelo, Erica Costantini and Antonio Di Stefano

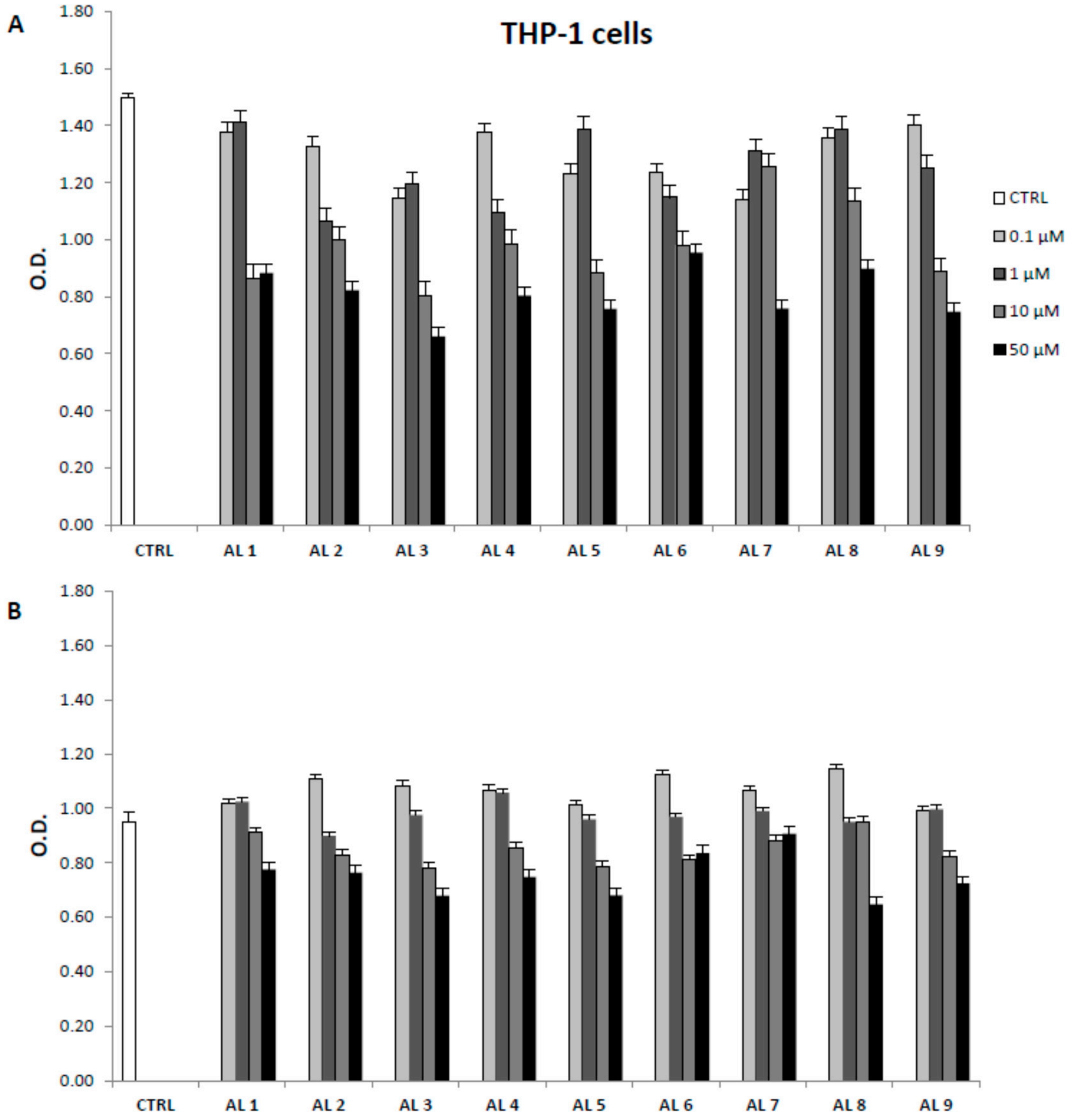

Figure S1. (**A**,**B**) Cellular vitality evaluated by MTT assay on THP-1 cell line for **AL1–9**.

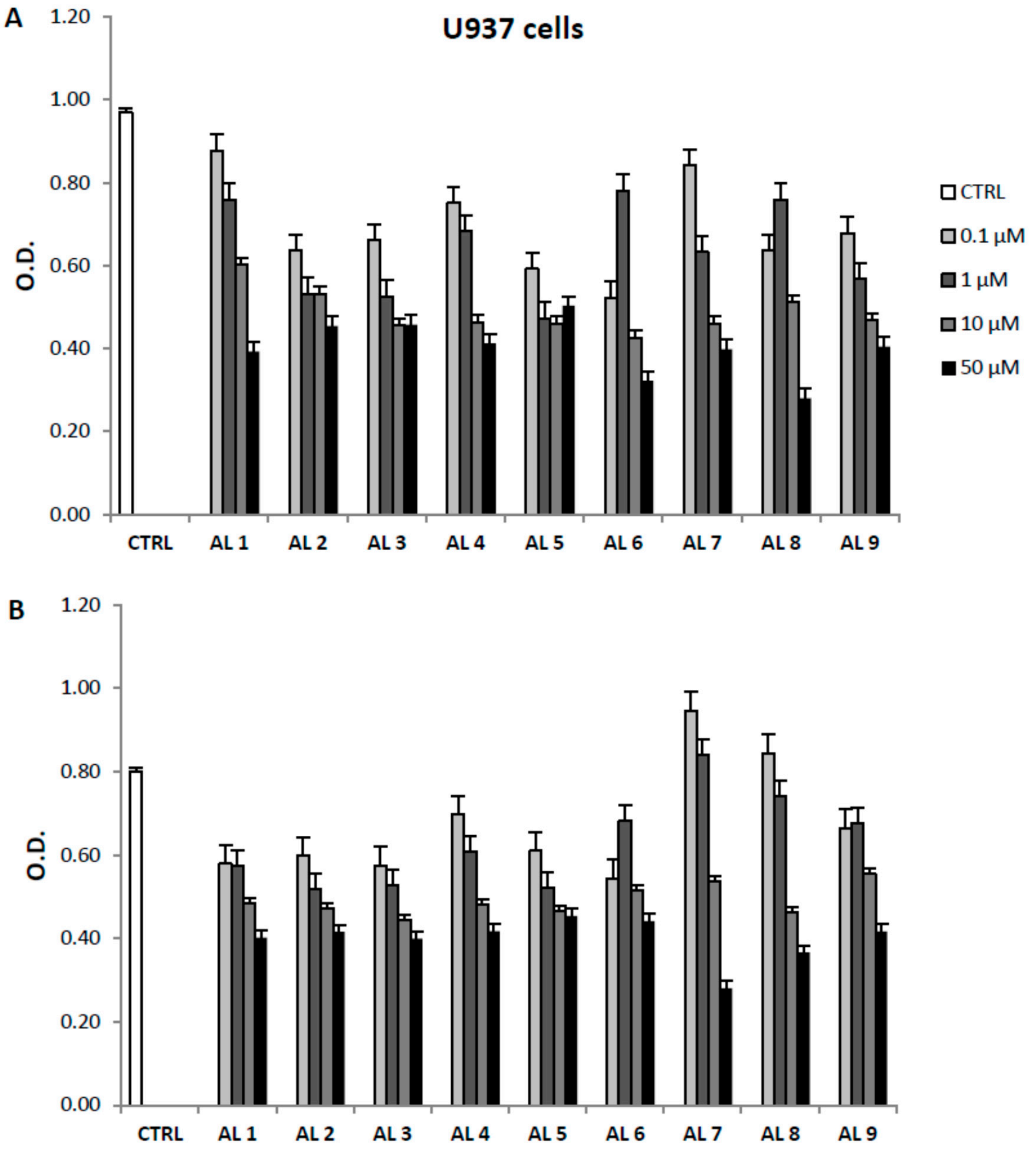

Figure S2. (**A**,**B**) Cellular vitality evaluated by MTT assay on U937 cell line for **AL1–9**.

(**A**)

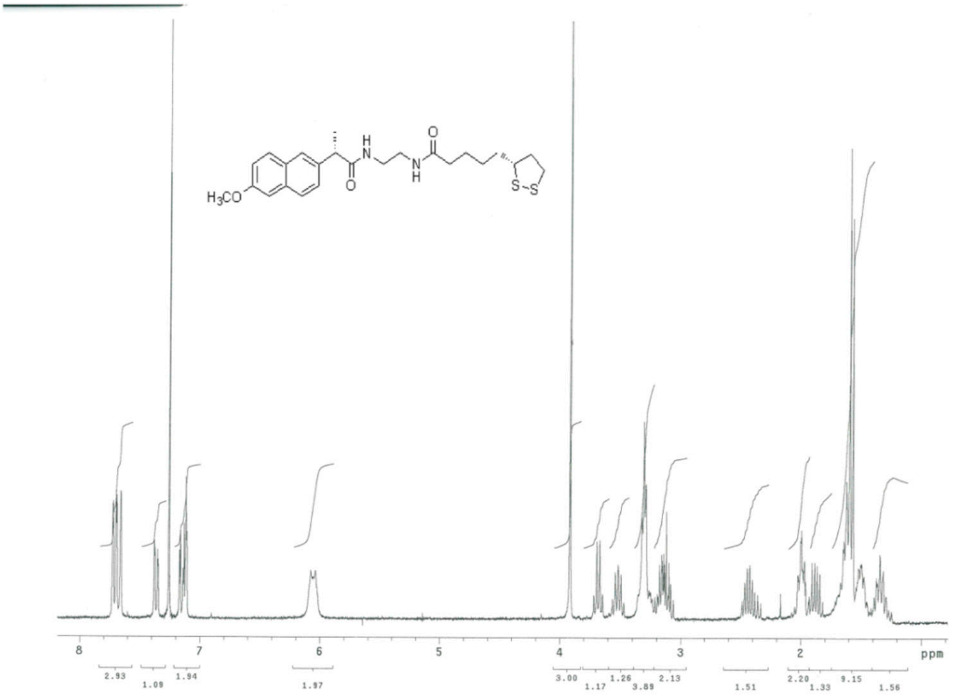

(**B**)

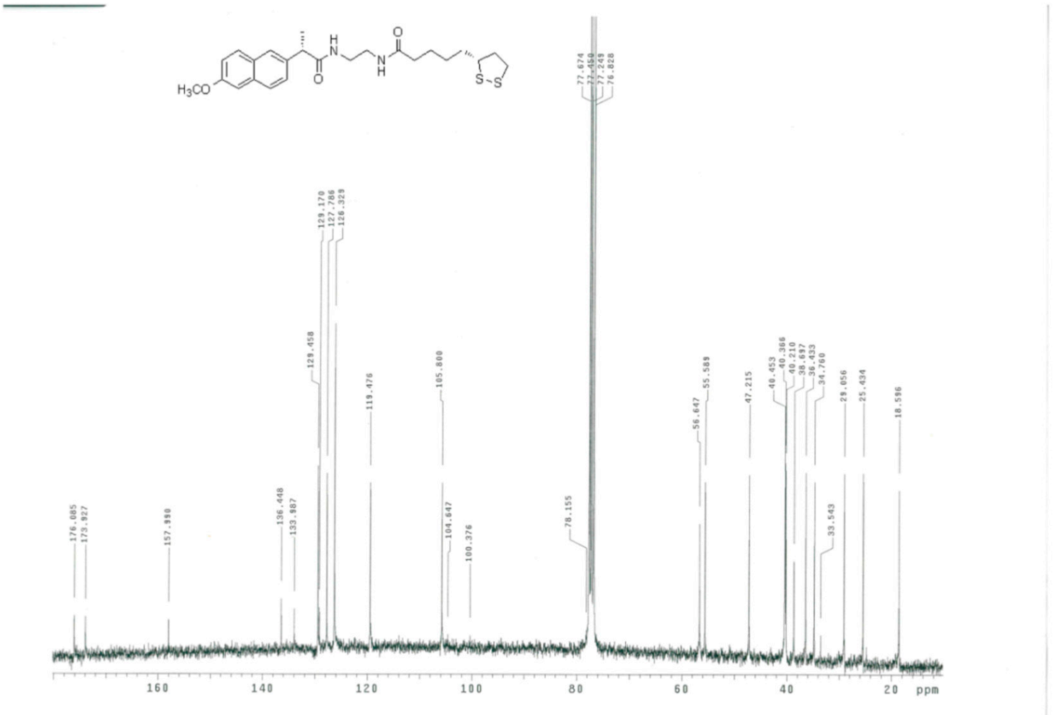

Figure S3. (**A**) ^{1}H- and (**B**) ^{13}C-NMR spectra of **AL4**.

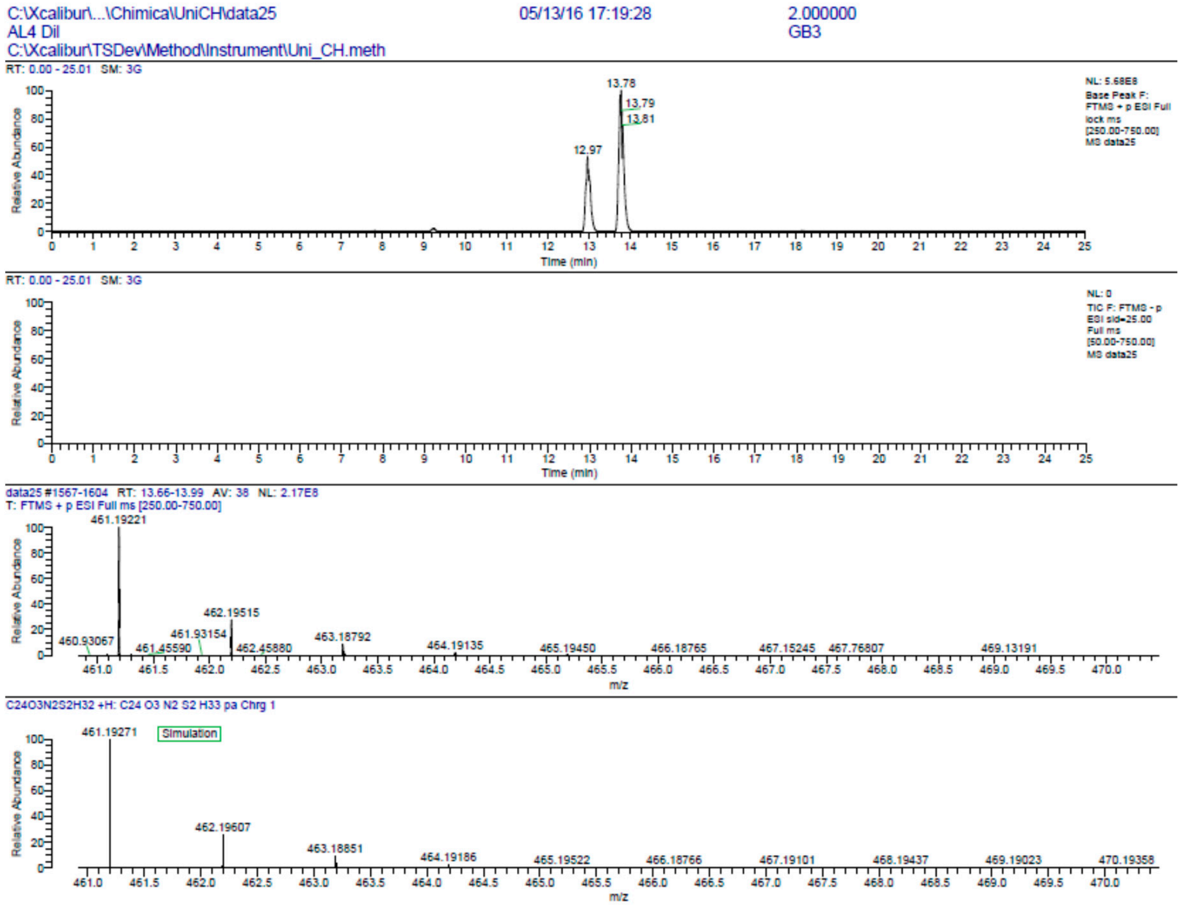

Figure S4. HR-MS spectra of **AL4**.

(**A**)

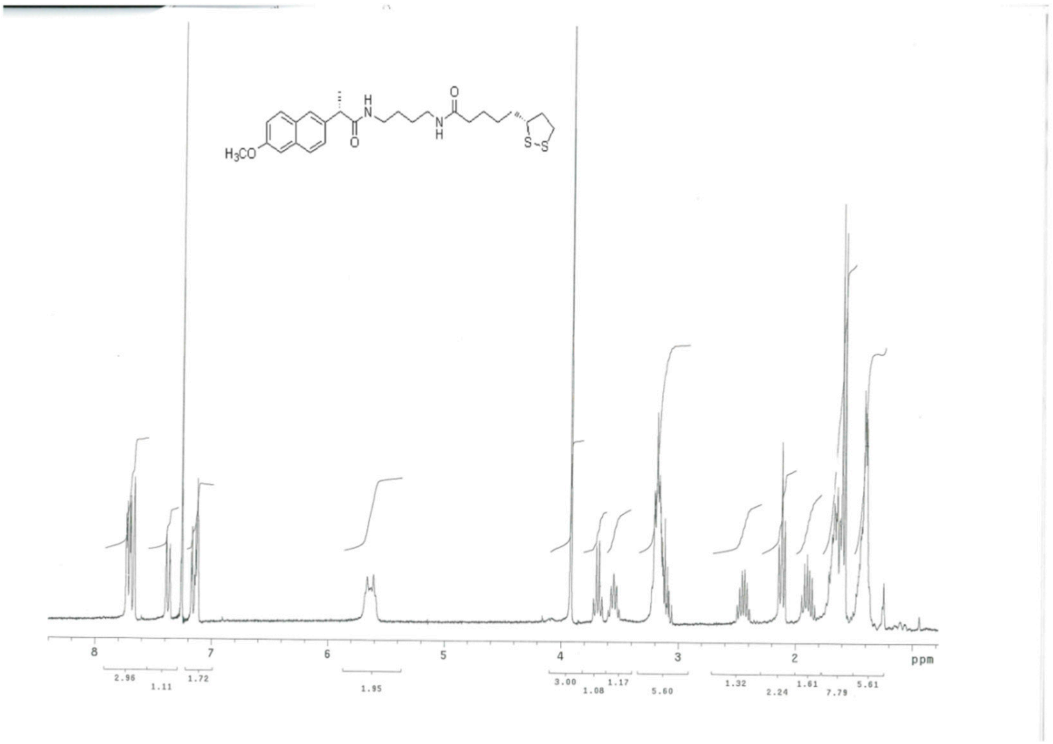

(**B**)

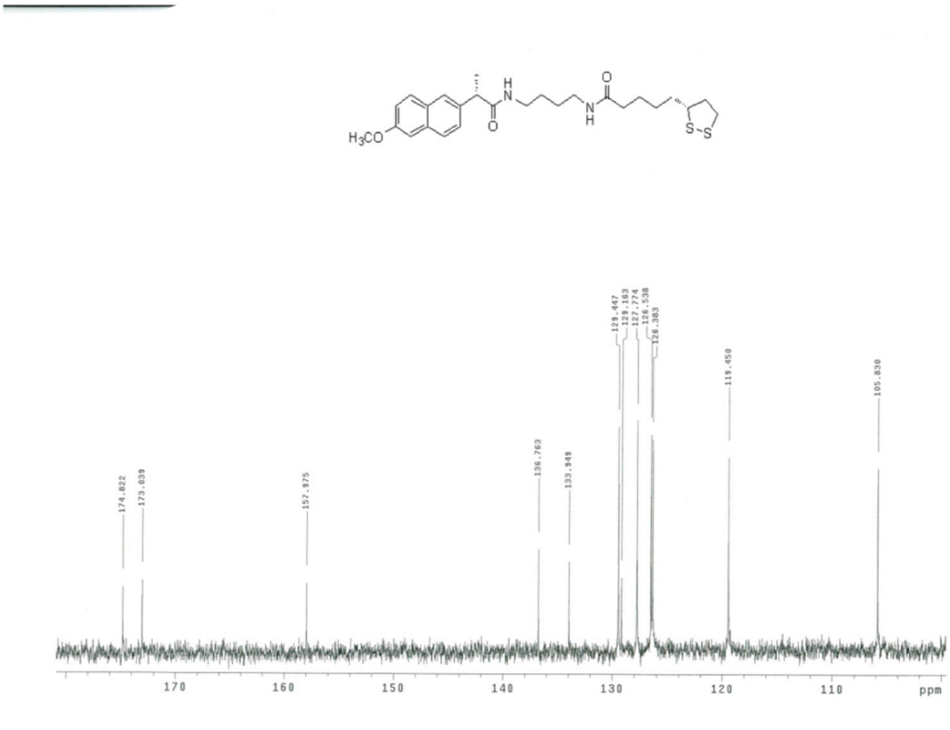

Figure S5. (**A**) ¹H- and (**B**) ¹³C-NMR spectra of **AL5**.

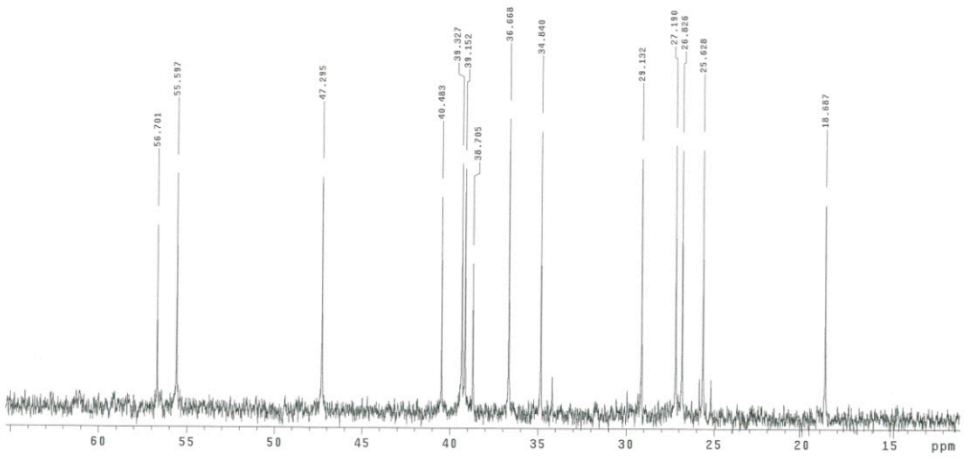

Figure S6. Expanded ^{13}C-NMR spectra of **AL5**.

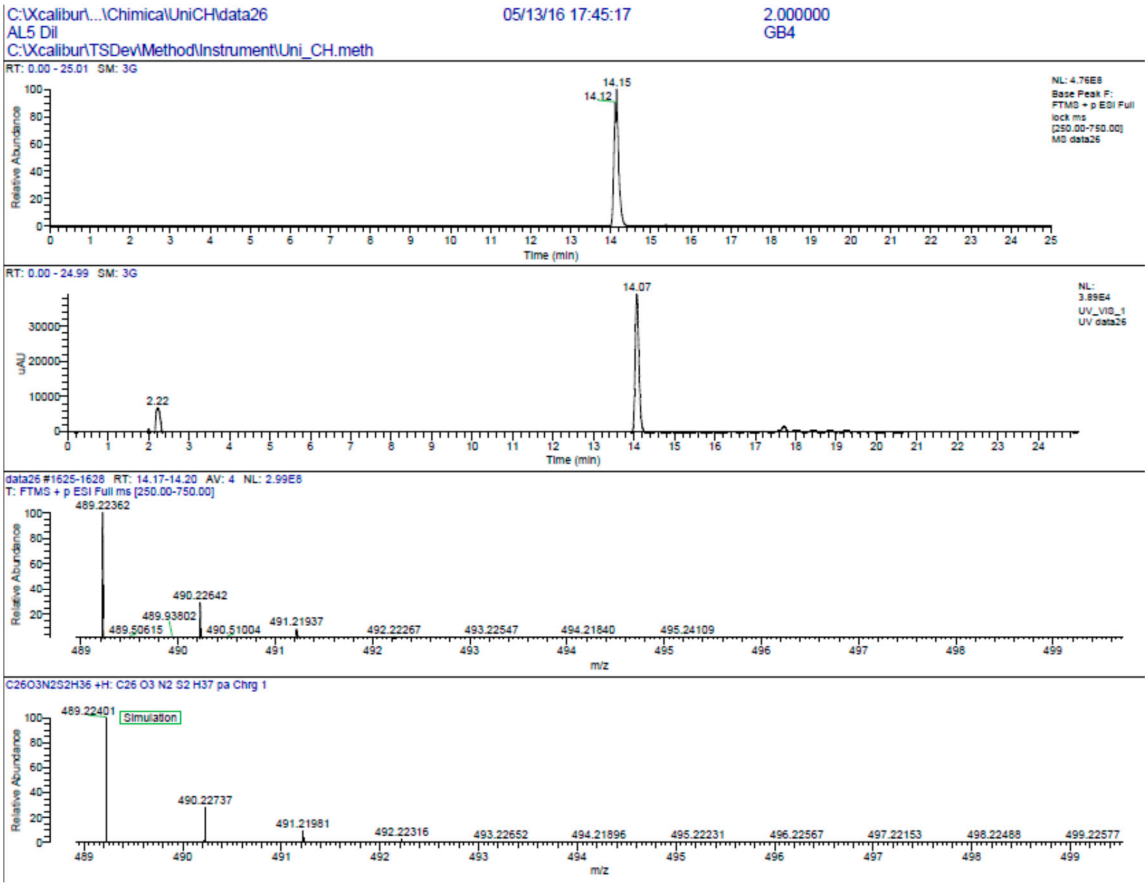

Figure S7. HR-MS spectra of **AL5**.

(**A**)

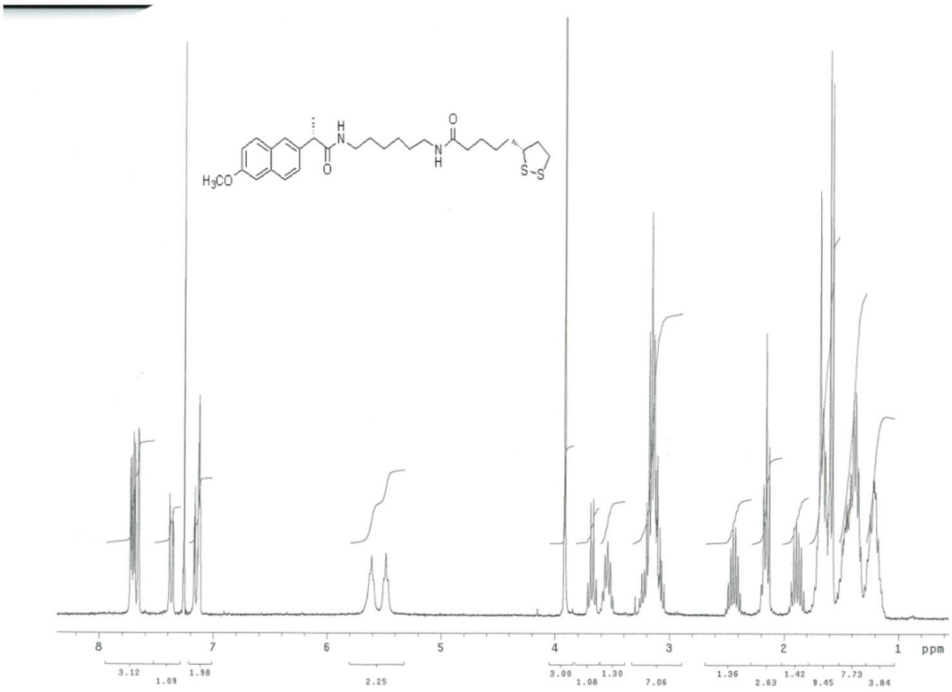

(**B**)

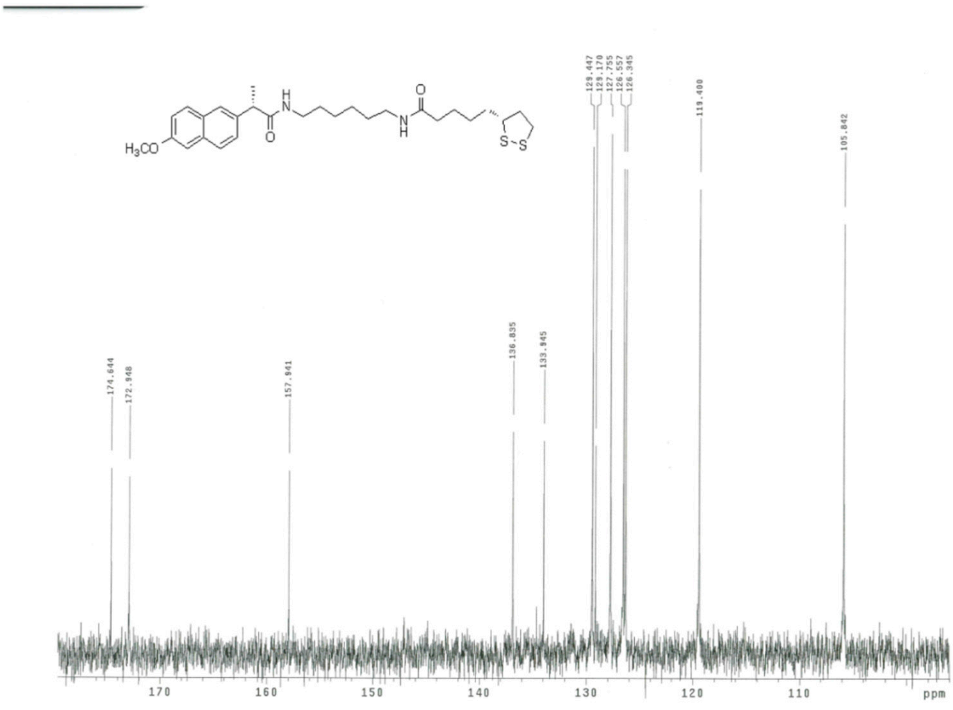

Figure S8. (**A**) ^{1}H- and (**B**) ^{13}C-NMR spectra of **AL6**.

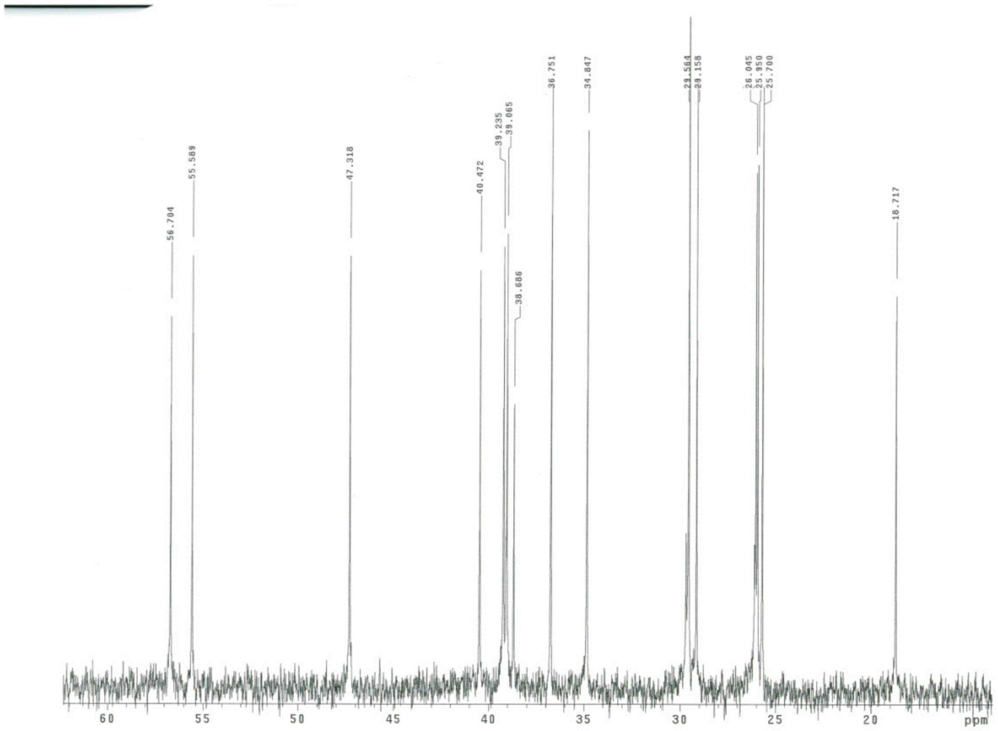

Figure S9. Expanded ^{13}C-NMR spectra of **AL6**.

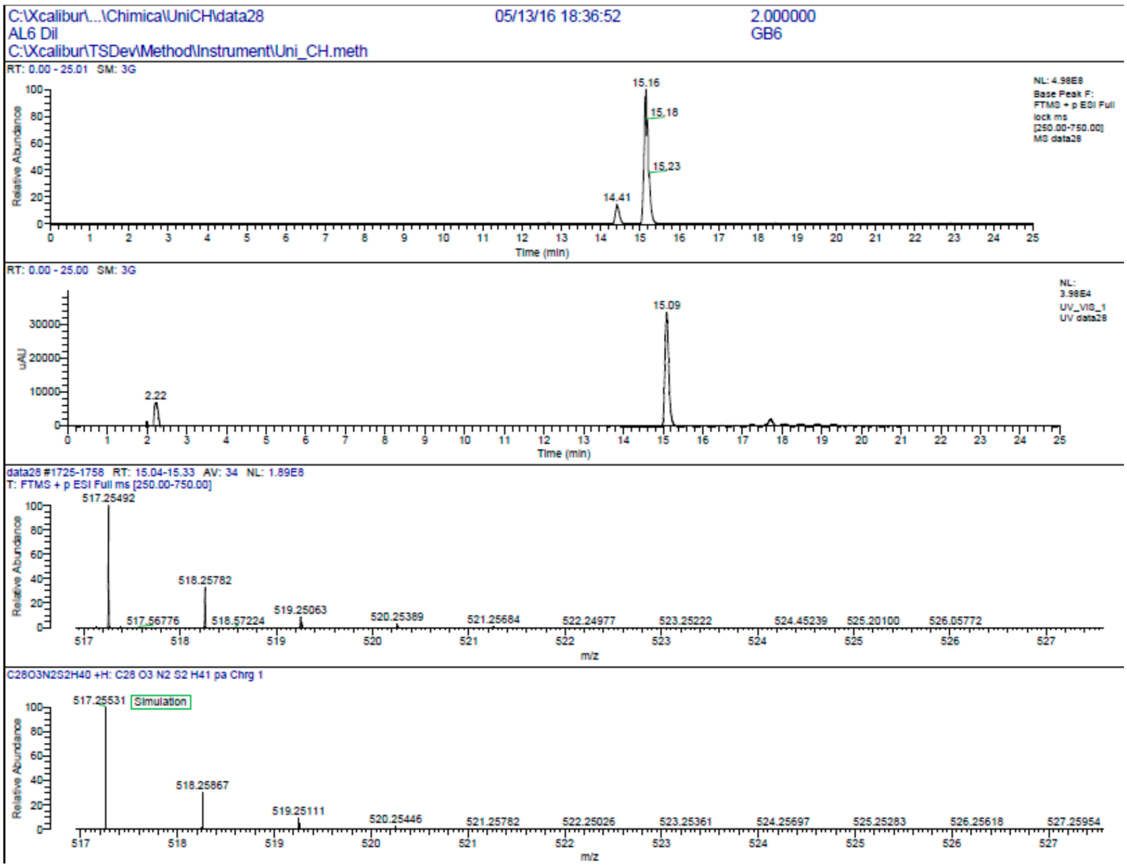

Figure S10. HR-MS spectra of **AL6**.

(**A**)

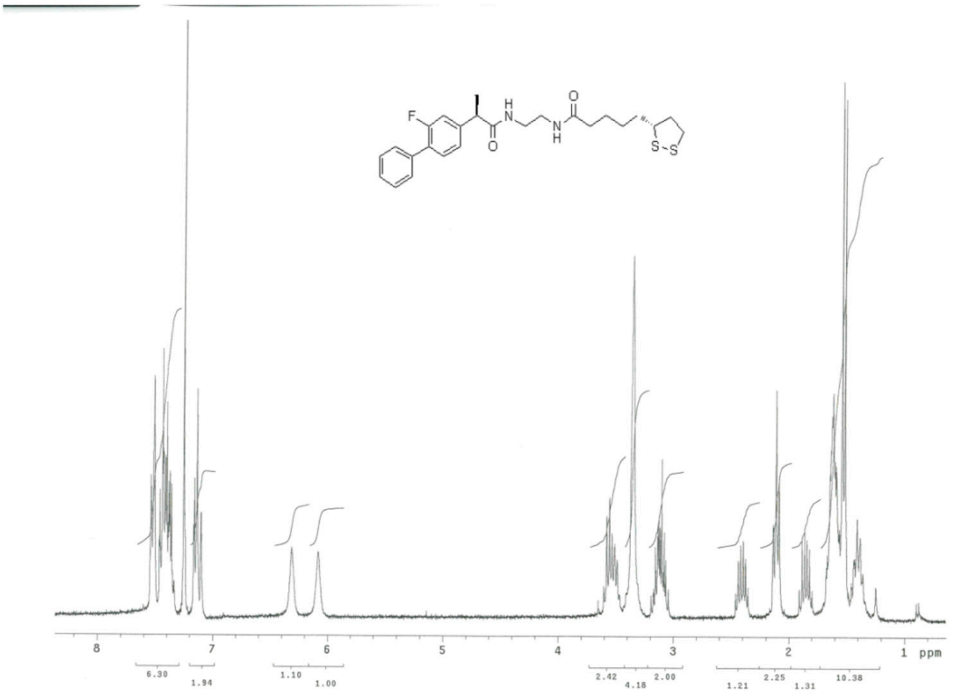

(**B**)

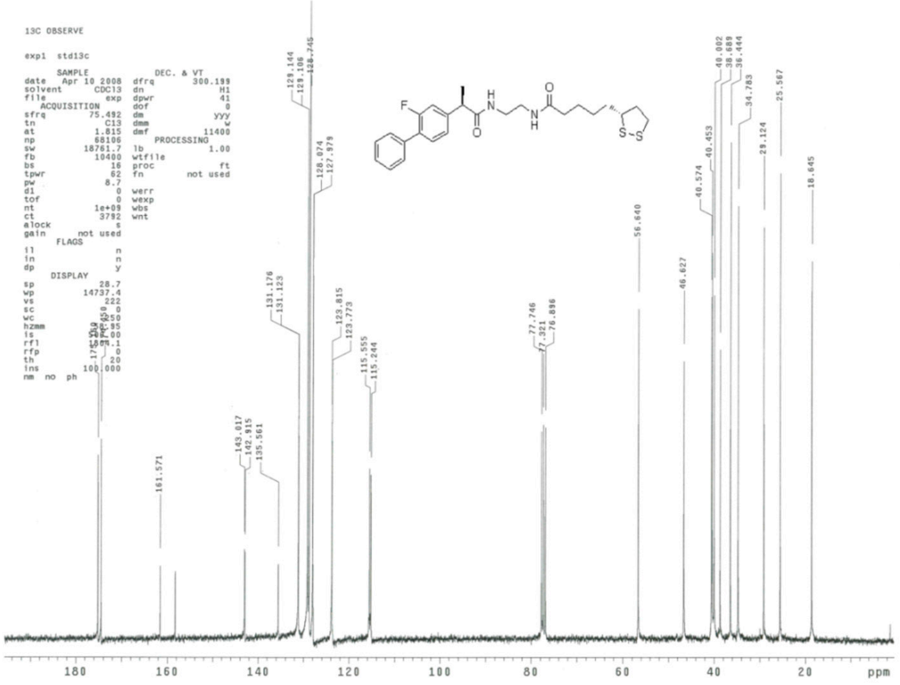

Figure S11. (**A**) ^{1}H- and (**B**) ^{13}C-NMR spectra of **AL7**.

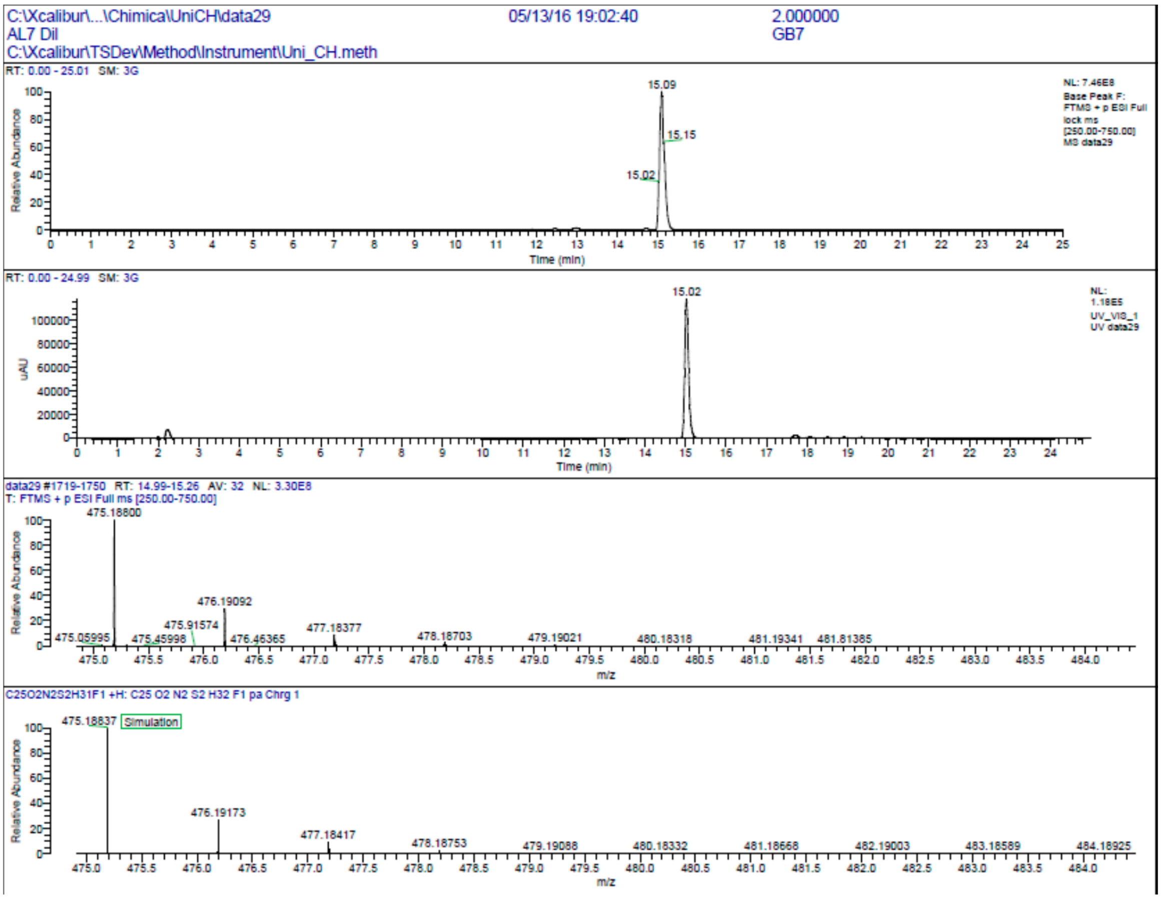

Figure S12. HR-MS spectra of **AL7**.

(**A**)

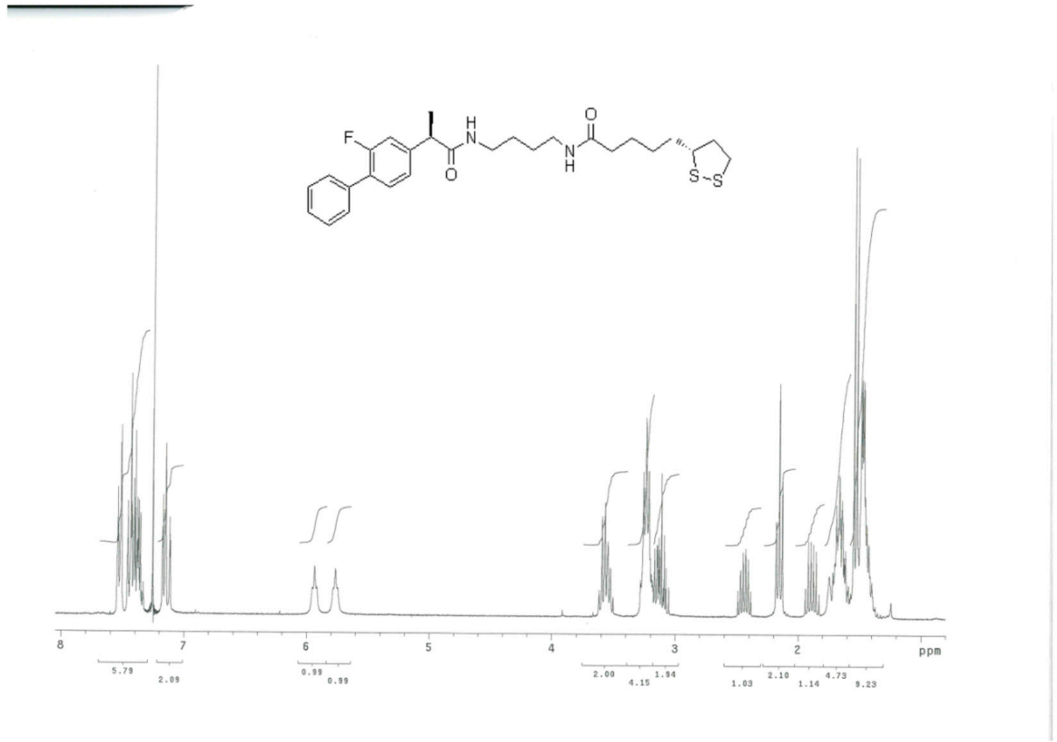

Figure S13. *Cont.*

(**B**)

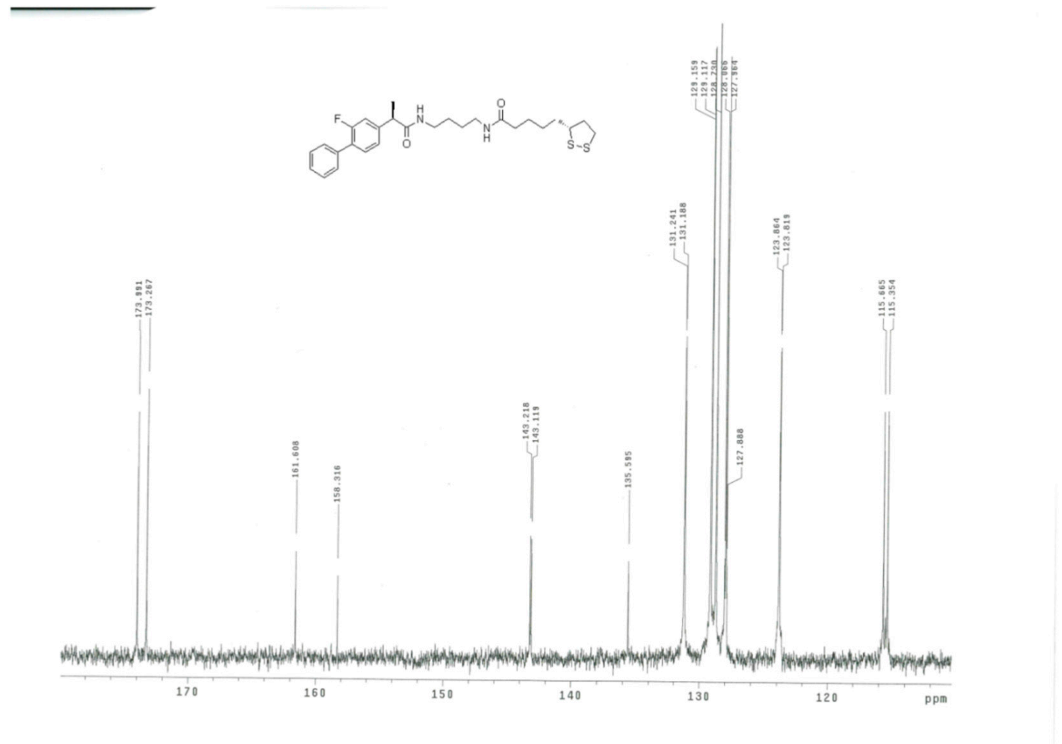

Figure S13. (**A**) ^{1}H- and (**B**)^{13}C-NMR spectra of **AL8**.

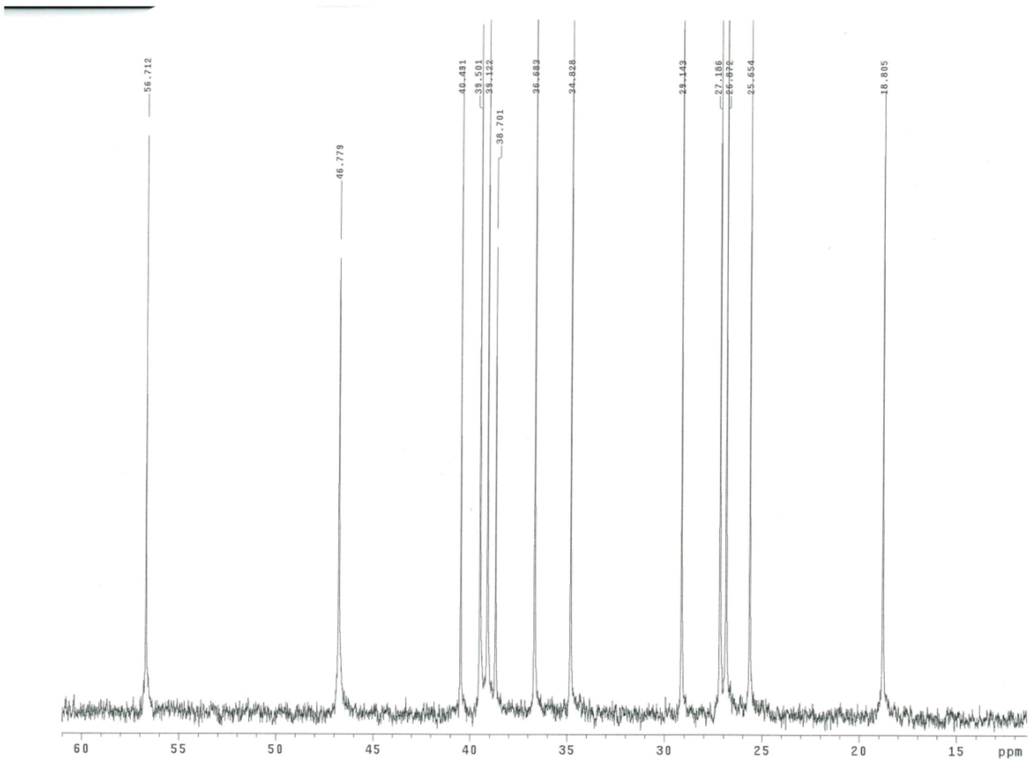

Figure S14. Expanded ^{13}C-NMR spectra of **AL8**.

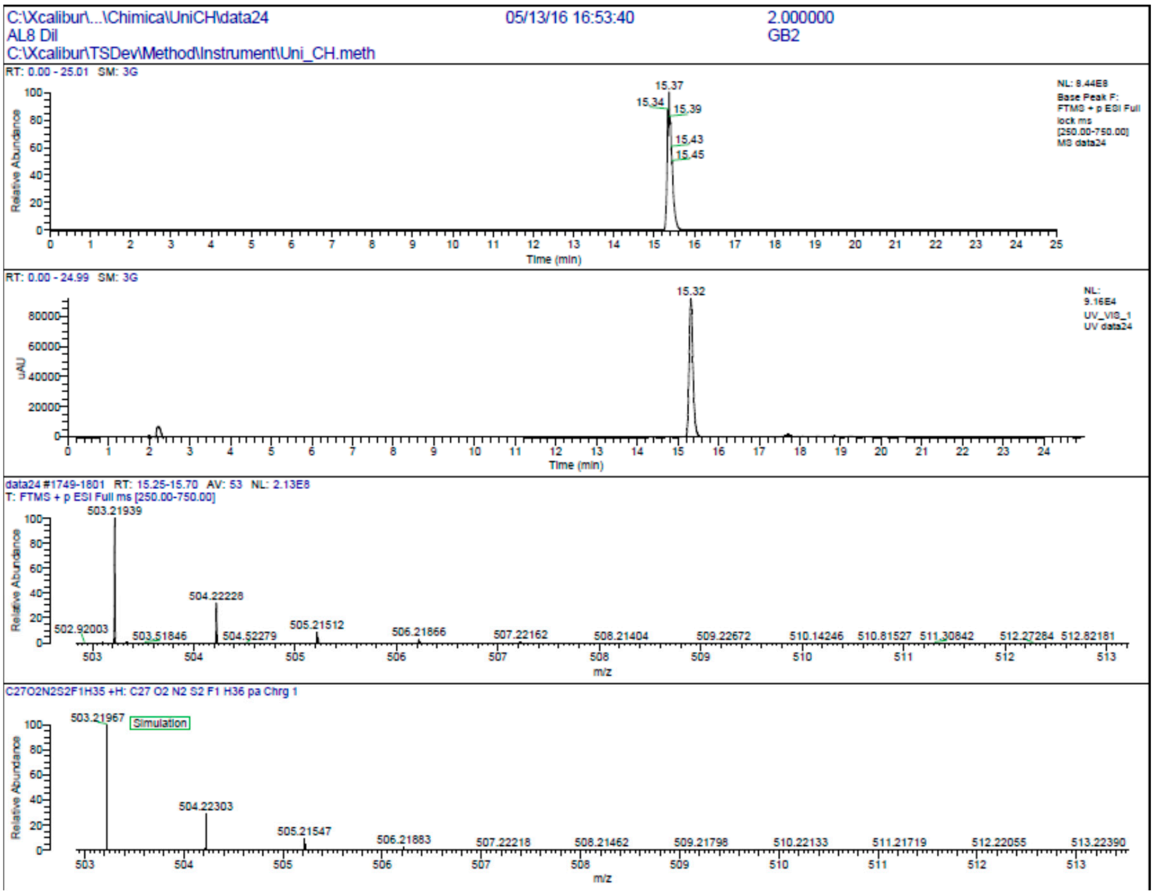

Figure S15. HR-MS spectra of **AL8**.

(**A**)

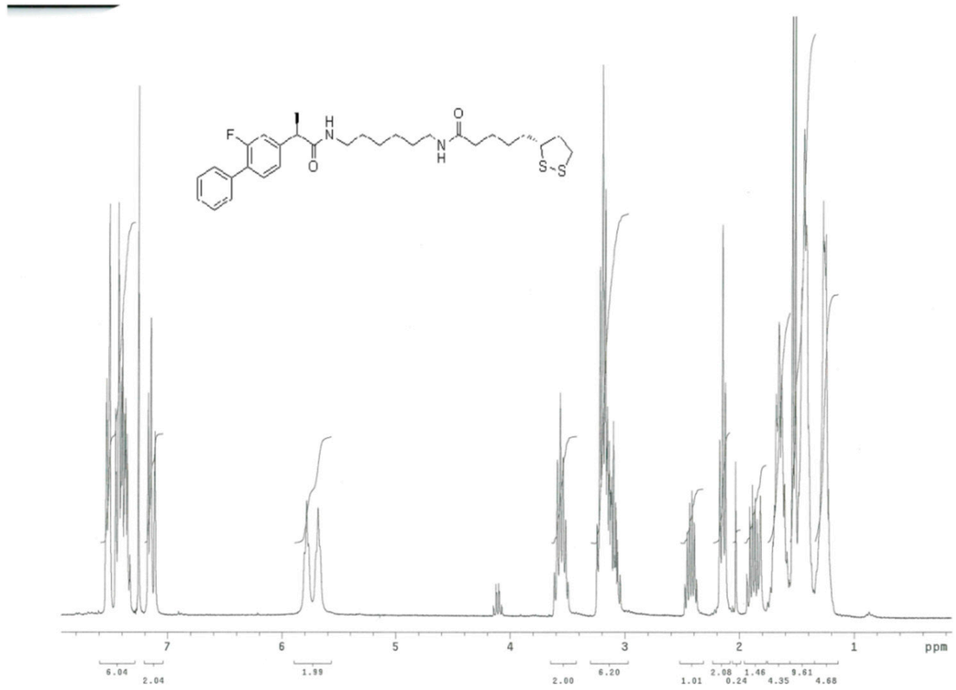

Figure S16. *Cont.*

(**B**)

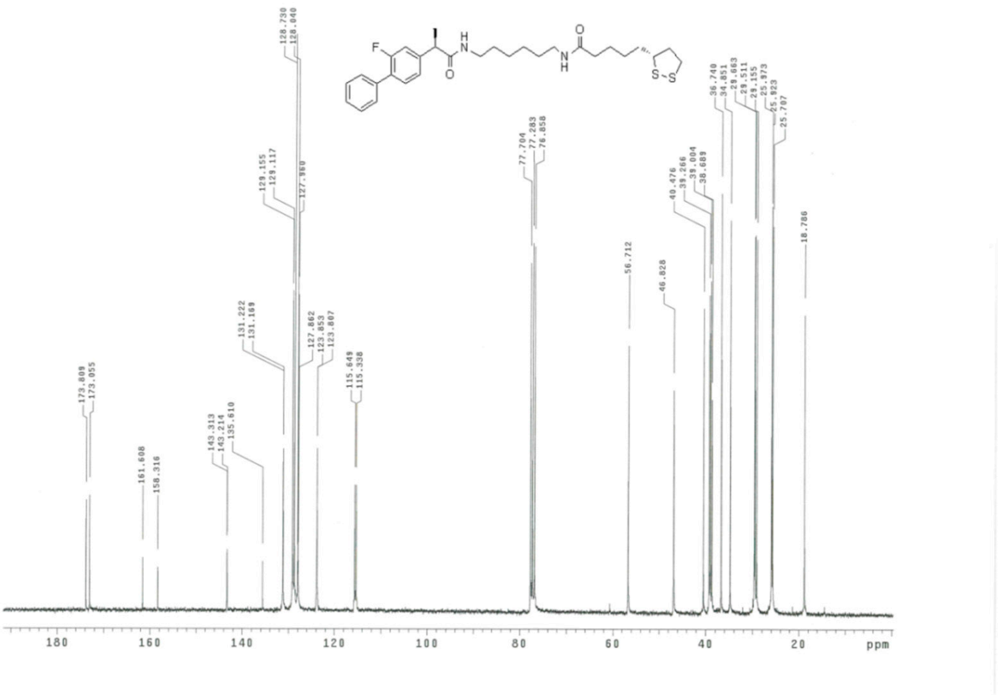

Figure S16. (**A**)^{1}H- and (**B**) ^{13}C-NMR spectra of **AL9**.

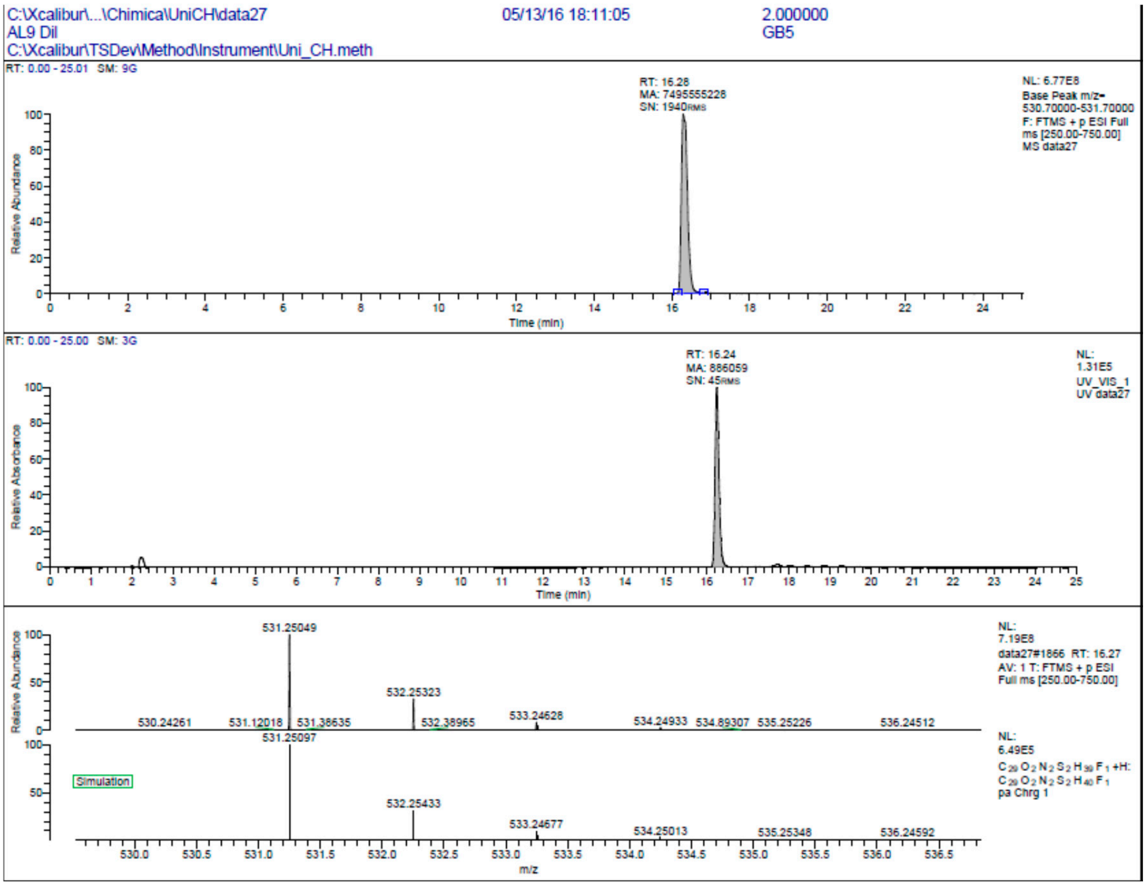

Figure S17. HR-MS spectra of **AL9**.

Table S1. Experimental conditions HR-MS.

Instrument Method: Uni_CH
Wednesday, 18 May 2016, 09:30:36; Page 1 of 4
Program for Dionex Chromatography MS Link
Column Oven. Temp Ctrl = On
Column Oven. Temperature. Nominal = 40.0 °C
Column Oven. Temperature. Lower Limit = 5.0 °C
Column Oven. Temperature. Upper Limit = 80.0 °C
Equilibration Time = None
Column Oven. Ready Temp Delta = 3.0 °C
Sampler. Temp Ctrl = Off
Pressure. Lower Limit = 10 bar
Pressure. Upper Limit = 468 bar
Maximum Flow Ramp Down = 0.250 mL/min^2
Maximum Flow Ramp Up = 0.250 mL/min^2
%A. Equate = "%A"
%B. Equate = "%B"
%C. Equate = "%C"
%D. Equate = "%D"
Draw Speed = 5.000 µL/s
Draw Delay = 3000 ms
Disp Speed = 20.000 µL/s
Dispense Delay = 0 ms
Waste Speed = 32.000 µL/s
Sample Height = 2.000 mm
Inject Wash = No Wash
Loop Wash Factor = 2.000
Puncture Offset = 0.0 mm
Pump Device = "Pump"
Inject Mode = Normal
Response Time = 2.000 s
UV_VIS_1.Wavelength = 280.0 nm
UV_VIS_1.Band width = 1 nm
UV_VIS_1.Ref Wavelength = Off
UV_VIS_1.Ref Band width = 1 nm
0.000 Wait UV. Ready and Pump. Ready and Column Oven. Ready and Sampler. Ready and Pump Module. Ready
Chromeleon sets this property to signal to Xcalibur that it is ready to start a run.
Ready To Run = 1
Xcalibur sets this property to start the run or injection.
Wait StartRun
Autozero
Flow = 0.200 mL/min
%B = 0.0%
%C = 20.0%
%D = 80.0%
Curve = 5
Wait UV. Ready and Pump. Ready and Column Oven. Ready and Sampler. Ready and Pump Module. Ready
Inject
Inject Response = 1
Chromeleon sets this property to signal the injection to Xcalibur.

Depending on your system configuration it might be necessary to manually insert a "Relay" command below in order to send the start signal to the MS.
Instrument Method: Uni_CH
Wednesday, 18 May 2016, 09:30:36; Page 2 of 4
Typical syntaxes:
Pump_Relay_1. Closed Duration = 2.00
UM3PUMP_Relay1. On Duration = 2.00
UV_VIS_1.AcqOn
Flow = 0.200 mL/min
%B = 0.0%
%C = 20.0%
%D = 80.0%
Curve = 5
15.000 Flow = 0.200 mL/min
%B = 0.0%
%C = 95.0%
%D = 5.0%
Curve = 5
20.000 Flow = 0.200 mL/min
%B = 0.0%
%C = 95.0%
%D = 5.0%
Curve = 5
Flow = 0.200 mL/min
%B = 0.0%
%C = 20.0%
%D = 80.0%
Curve = 5
24.000 Flow = 0.200 mL/min
%B = 0.0%
%C = 20.0%
%D = 80.0%
Curve = 5
25.000 UV_VIS_1. AcqOff
Inject Response = 0
End
Instrument Method: Uni_CH
Wednesday, 18 May 2016, 09:30:36; Page 3 of 4
Method of Q Exactive
Overall method settings
Global Settings
use lock masses best
Lock mass injection—
Chrom. peak width (FWHM) 30 s
Time
Method duration 25.00 min
Customized Tolerances (+/−)
Inclusion
Exclusion
Neutral Loss
Mass Tags
Dynamic Exclusion

Experiment
Full MS—SIM
General
Runtime 0 to 25 min
Polarity Positive
In-source CID 0.0 eV
Full MS—SIM
Microscans 1
Resolution 140,000
AGC target 5 e 5
Maximum IT 200 ms
Number of scan ranges 1
Scan range 250 to 750 m/z
Spectrum data type Profile
Setup
Tunefiles
General
Switch Count 0
Base Tunefile C:\Xcalibur\TSDev\Method\cal mix.mstune
Contact Closure
General
Used False
Start in Closed True
Switch Count 0
Syringe
General
Instrument Method: Uni_CH
Wednesday, 18 May 2016, 09:30:36; Page 4 of 4
Used False
Start in OFF True
Stop at end of run False
Switch Count 0
Pump setup
Syringe type Hamilton
Flow rate 3.000 μL/min
Inner diameter 2.303 mm
Volume 250 μL
Divert Valve A
General
Used False
Start in 1-2 True
Switch Count 0
Divert Valve B
General
Used False
Start in 1-2 True
Switch Count 0
Lock Masses
1 entry
Mass Polarity Start End Comment
